# MXene molecular sieving membranes for highly efficient gas separation

**DOI:** 10.1038/s41467-017-02529-6

**Published:** 2018-01-11

**Authors:** Li Ding, Yanying Wei, Libo Li, Tao Zhang, Haihui Wang, Jian Xue, Liang-Xin Ding, Suqing Wang, Jürgen Caro, Yury Gogotsi

**Affiliations:** 1 School of Chemistry and Chemical Engineering, South China University of Technology, 510640 Guangzhou, China; 2 Institute of Physical Chemistry and Electrochemistry, Leibniz University of Hannover, Callinstrasse 3A, 30167 Hannover, Germany; 3 Department of Materials Science and Engineering, and A. J. Drexel Nanomaterials Institute, Drexel University, Philadelphia, PA 19104 USA; 4 Key Laboratory of Physics and Technology for Advanced Batteries (Ministry of Education), College of Physics, Jilin University, 130012 Changchun, China

## Abstract

Molecular sieving membranes with sufficient and uniform nanochannels that break the permeability-selectivity trade-off are desirable for energy-efficient gas separation, and the arising two-dimensional (2D) materials provide new routes for membrane development. However, for 2D lamellar membranes, disordered interlayer nanochannels for mass transport are usually formed between randomly stacked neighboring nanosheets, which is obstructive for highly efficient separation. Therefore, manufacturing lamellar membranes with highly ordered nanochannel structures for fast and precise molecular sieving is still challenging. Here, we report on lamellar stacked MXene membranes with aligned and regular subnanometer channels, taking advantage of the abundant surface-terminating groups on the MXene nanosheets, which exhibit excellent gas separation performance with H_2_ permeability >2200 Barrer and H_2_/CO_2_ selectivity >160, superior to the state-of-the-art membranes. The results of molecular dynamics simulations quantitatively support the experiments, confirming the subnanometer interlayer spacing between the neighboring MXene nanosheets as molecular sieving channels for gas separation.

## Abstract (web summary)

Two-dimensional materials show great potential for membrane technologies, but their disordered channels hinder their molecular sieving performance. Here, Wang, Gogotsi and colleagues design a MXene membrane with ordered nanochannels that exhibits an excellent H_2_/CO_2_ gas separation performance.

## Introduction

Gas separation with membrane technology is attractive because of its high efficiency, low energy consumption, and simple operation^1–3^. Membranes with high permeability and high selectivity are urgently required^3^. The recent use of two-dimensional (2D) materials^4,5^, such as graphene and graphene oxide (GO)^6–13^, zeolite or metal–organic framework (MOF) nanosheets^14–16^, has led to innovative membrane designs. Previous studies have shown that MOF nanosheets are promising for membrane assembly^15,16^ and a pioneering breakthrough work on zeolite nanosheets based membrane was also conducted by Tsapatsis^14,17–19^, where the molecules were mainly transported through the intrinsic pores in the 2D nanosheets. But the types of zeolite or MOFs that can be easily exfoliated are rather limited due to the structural deterioration in exfoliation process^15,16^. Similarly, the monolayer graphene with artificial sub-nanopores created by selective etching or ion bombardment is emerged as selective membrane for gas separation or ion sieving^20–22^. However, it is difficult to fabricate the graphene sheets with controllable and uniform pores due to the stochastic nature, which limits the industrial applications. In contrast to the membranes with intrinsic or artificial pores on the nanosheets as the main molecular sieving channels, another kind of 2D laminar membrane has attracted increasing attention due to its simple preparation and easy to large-scale fabrication, in which the molecules are transported and sieved through the interlayer nanochannels between the neighboring nanosheets^6–8,23^. Therefore, for the latter 2D laminar membranes, the stacking structure of the nanosheets strongly affects the separation performance^6–8^. For instance, a GO membrane with randomly stacked structure exhibited only Knudsen diffusion during gas separation, while a membrane with an ordered structure exhibited molecular sieving with a greatly increased gas separation factor^6^. Moreover, many other well-ordered GO laminates exhibited enhanced gas or water separation performance in terms of their selectivity and permeability compared to the disordered ones^7,8^. However, since the oxygen-containing functional groups that decorate the defects in GO sheets are difficult to control, random laminar structures are easily formed when such sheets are stacked into membranes^7,8^. Another young family of 2D materials named “MXenes” with the formula of M_*n*+1_X_*n*_T_X_, are usually produced by selectively etching the A-group (mainly group IIIA or IVA elements) layers from M_*n*+1_AX_*n*_ phases (*n* = 1, 2, or 3), where M is an early transition metal and X is carbon and/or nitrogen. More importantly, abundant of surface-terminating groups (T_X_: = O, –OH and –F) are formed evenly on the entire surface of the nanosheets during the etching and delaminating processes^24–31^. Interestingly, the variety of T_X_ species can create open narrow nanochannels between the neighboring nanosheets in stacked MXene laminates, making MXene a promising material to assemble highly efficient membranes^27^.

Here, exfoliated MXene nanosheets were used as building blocks to construct 2D laminated membranes for selective gas separation for the first time, as demonstrated using a model system of H_2_ and CO_2_. The MXene membranes exhibit excellent performance in terms of the hydrogen permeability and H_2_/CO_2_ selectivity, transcending the state-of-the-art membranes. Such high-permeability hydrogen-selective membranes are desired in many fields, such as hydrogen production and carbon dioxide capture.

## Results

### Preparation of MXene nanosheets

**Fig. 1:**
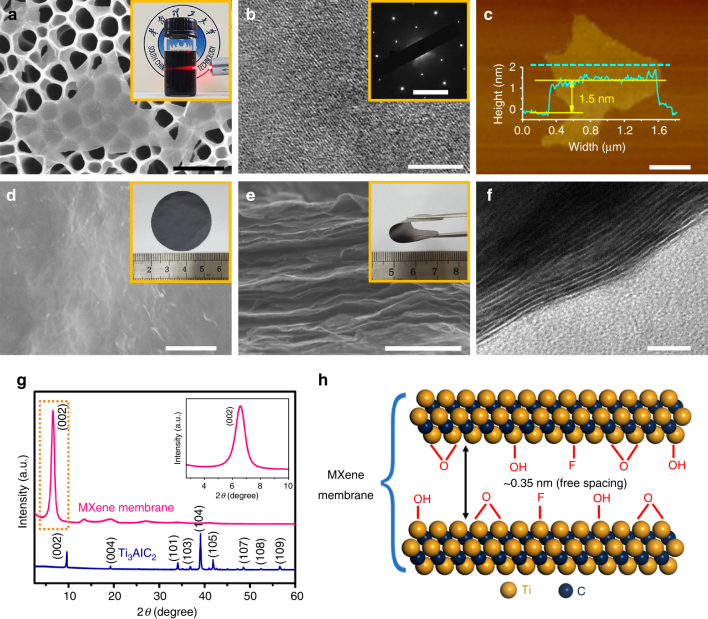
Morphology and structure of exfoliated MXene (Ti_3_C_2_T_X_) nanosheets and stacked MXene membrane. **a** SEM image of the delaminated MXene nanosheets on porous anodic aluminum oxide (AAO) (scale bar, 1 μm). Inset is the Tyndall scattering effect in MXene colloidal solution in water. **b** HRTEM image of the MXene nanosheet with SAED pattern in the inset (scale bar, 5 nm, inset **b**, 5 nm^−1^). **c** AFM image of the MXene nanosheet on cleaved mica. The height profile of the nanosheet corresponds to the blue dashed line (scale bar, 500 nm). Note that the adsorbed molecules, such as H_2_O, also contribute the detected thickness of 1.5 nm. **d** SEM image of the MXene membrane surface (scale bar, 500 nm). Inset is a photograph of a MXene membrane. **e** Cross-sectional SEM image of the MXene membrane (scale bar, 1 μm). Inset is a tweezer bent membrane. **f** Cross-sectional TEM image of the MXene membrane with 2D channels (scale bar, 10 nm). **g** XRD patterns of the MAX (Ti_3_AlC_2_) powder and MXene (Ti_3_C_2_T_X_) membrane with inset of the magnified XRD pattern at low Bragg angles. **h** Illustration of the spacing between the neighboring MXene nanosheets in the membrane

The most common MXene, Ti_3_C_2_T_X_, is obtained after selectively etching Al from the corresponding MAX (Ti_3_AlC_2_) phase using hydrochloric acid and lithium fluoride^24–26,29^, the structures are displayed in Supplementary Fig. 1 and explained in Supplementary Note 1. The Tyndall scattering effect in the as-prepared MXene colloidal suspension is clearly observed (Fig. 1a, inset, Supplementary Fig. 2, and Supplementary Note 2). The scanning electron microscopy (SEM) and transmission electron microscopy (TEM) images (Fig. 1a and Supplementary Fig. 3) show that the exfoliated MXene nanosheets are very thin and nearly transparent to the electron beams. High-resolution TEM (HRTEM) image and selected-area electron diffraction (SAED) patterns (Fig. 1b and Supplementary Figs. 4 and 5) indicate the hexagonal structure of the basal planes and high crystallinity of the MXene flakes without obvious nanometer-scale defects or carbide amorphization. As indicated from the atomic force microscopy (AFM) measurements (Fig. 1c and Supplementary Fig. 6), most of the MXene nanosheets have a uniform thickness of 1.5 nm with a lateral size of 1–2 μm. Considering that the theoretical thickness of a Ti_3_C_2_T_X_ single layer is ~1 nm^29,32^, and MXene nanosheets adsorb water and other molecules that also contribute to the total thickness, the 1.5-nm-thick nanosheet should be monolayer Ti_3_C_2_T_X_^26,32^.

### Preparation of 2D MXene membranes

The MXene membranes were fabricated using vacuum-assisted filtration on anodic aluminum oxide (AAO) support (Fig. 1a and Supplementary Fig. 7). After detaching the MXene layers from the substrate, free-standing MXene membranes were directly obtained with good flexibility (Fig. 1e and Supplementary Figs. 8 and 9). From the top-view SEM and AFM images (Fig. 1d and Supplementary Fig. 10), the membrane is determined to be intact, and the terminating groups were also detected on the MXene membrane (see Supplementary Figs. 11–17 and Supplementary Tables 1–3 for the Fourier transform infrared spectroscopy (FTIR), thermogravimetric analysis (TGA), energy dispersive X-ray spectroscopy (EDX), and X-ray photoelectron spectroscopy (XPS) results). The cross-sectional SEM image and elemental distribution (Fig. 1e and Supplementary Figs. 13–15) indicate a homogeneous laminar structure throughout the membrane. The cross-sectional TEM images (Fig. 1f and Supplementary Fig. 18) reveal well-organized, highly ordered subnanometer channels resulting from the evenly distributed terminating groups on the MXene nanosheet surface^30,33,34^. The sharp (002) peak with high intensity in the powder X-ray diffraction (XRD, Fig. 1g) results further confirms the ordered stacking in the MXene membrane. The (002) peak at 2*θ* = 6.6° indicates the *d*-spacing of ~1.35 nm, based on Bragg’s law (Supplementary Fig. 19, Supplementary Note 3, and Supplementary Equation (2)). After deducting the monolayer thickness of ~1 nm^29,32^, the free spacing between the neighboring MXene nanosheets is estimated to be ~0.35 nm (Fig. 1h), which could serve as a molecular sieve to separate gases by membrane permeation.

### Gas separation performance of 2D MXene membranes

**Fig. 2:**
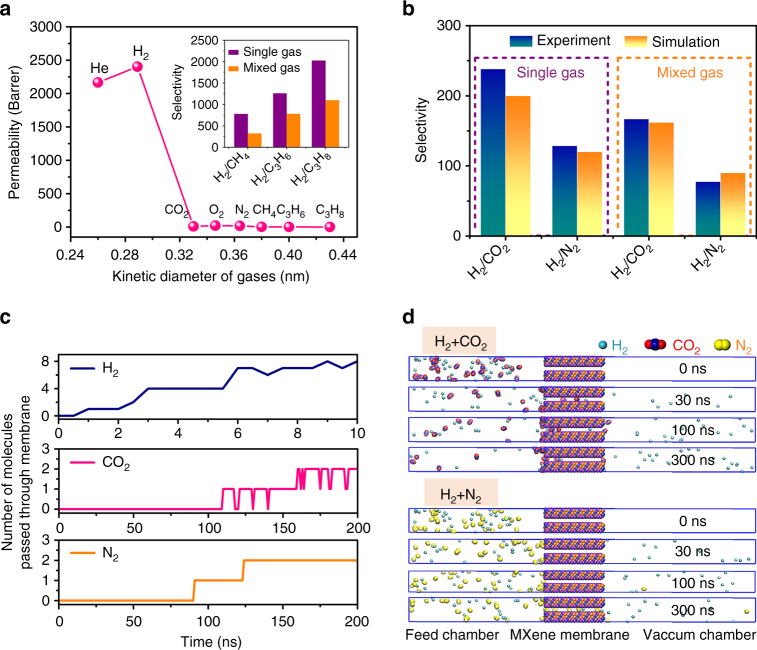
MD simulations of the gas permeation through the MXene membrane compared with the experimental results. **a** Single-gas permeabilities through a 2-μm-thick MXene membrane as a function of the gas kinetic diameter at 25 °C and 1 bar. Inset shows the selectivity of H_2_ relative to the other gases in both the single-gas and equimolar mixed-gas permeation studies. **b** Comparison of the experimental and MD simulated selectivities of H_2_/CO_2_ and H_2_/N_2_ in both the single-gas and mixed-gas permeations. **c** The number of gas molecules that passed through the MXene membrane in MD simulation as a function of simulation time for single-gas permeation. For H_2_, only the first 10 ns of the simulation are shown because of its fast permeation. By contrast, only two molecules pass through the MXene membrane during the 200-ns-long simulation for CO_2_ or N_2_. The CO_2_ curve fluctuates because of CO_2_ adsorption–desorption on the MXene membrane. **d** Simulation snapshots at 0, 30, 100, and 300 ns for two sets of mixed-gas permeation systems: (H_2_ + CO_2_) and (H_2_ + N_2_). Note that H_2_ was modeled by united-atom force field. The MXene membrane was composed of two nanosheets with a free spacing of 0.35 nm located in the middle of the simulation system. In the beginning (*t* = 0 ns), 30 H_2_ and 30 CO_2_ (or N_2_) molecules were present in the feed chamber, which permeated through the MXene membrane to the evacuated permeate chamber. The details can be found in section of “Methods”

The MXene membranes were sealed into Wicke–Kallenbach permeation cells to measure the gas separation performance (Supplementary Figs. 20 and 21). For our MXene membrane, the permeability of the small gas molecules (2164 Barrer for He and 2402 Barrer for H_2_) is much higher than that of the gases with bigger kinetic diameters (Fig. 2a and Supplementary Table 4), showing a clear cutoff in between. The ideal selectivity (238.4) of the single-gas permeation and the separation factor (166.6) of the mixed-gas permeation of H_2_/CO_2_ are much higher than the corresponding Knudsen coefficient (4.7). Obviously, the gas permeation is mainly dominated by the gas kinetic diameter rather than its molecular weight (Fig. 2a and Supplementary Fig. 22), known as the molecular sieving (size exclusion) mechanism. Very interestingly, the permeability of CO_2_ (10 Barrer) is approximately half of N_2_ (19 Barrer), although its kinetic diameter (0.33 nm) is 9% smaller than that of N_2_ (0.364 nm). Here, adsorption modifies the molecular sieving process. Because CO_2_ has a much larger quadrupole moment than N_2_, it interacts with the MXene membrane stronger (the interaction energy values of MXene with CO_2_ and N_2_, as calculated by molecular dynamics (MD) simulations, are −175.1 and −97.5 kJ mol^−1^, respectively, see Supplementary Note 4 and 5), which considerably suppress the CO_2_ diffusion in the MXene subnanometer channels^6,7^. The adsorption isotherms of the gases on the MXene membranes at 25 °C also indicate a preferential adsorption of CO_2_ compared to N_2_ or other gases (Supplementary Fig. 23), even though the adsorption capacities of the MXene nanosheets are quite small^15,16,35^. The adsorbed CO_2_ molecules in the subnanochannels can even block the passing molecules and increase the resistance to CO_2_ diffusion, while such phenomenon is absent for H_2_, resulting a high separation factor of H_2_/CO_2_. For O_2_, its kinetic diameter (0.346 nm) is just slightly smaller than the interlayer spacing of the MXene membrane (0.35 nm). Although O_2_ can pass through the subnanochannels in the membrane, but with a much larger mass transfer resistance due to the confinement of the neighboring MXene nanosheets. That is why the O_2_ permeability is significantly lower than that of much smaller molecules, such as He and H_2_.

### Gas separation mechanism

To elucidate the gas separation mechanism, two sets of atomistic MD simulations (total simulation time >5 μs) were performed to study the gas transport through the MXene membrane, as schematically shown in Supplementary Fig. 24^16,36^. First, the confined diffusion coefficients of He, H_2_, CO_2_, O_2_, N_2_, and CH_4_ in two neighboring MXene channels with 0.35 nm free spacing were calculated by MD simulations (Supplementary Fig. 25 and Supplementary Note 4)^37^. The simulation yields a diffusivity ratio of 175:238:1.0:4.1:1.4:0.1. Furthermore, hundreds-nanosecond (ns)-long MD simulations were carried out to study the passage of the gas molecules through the MXene membrane^16,36^. In simulations of single-gas permeation (Fig. 2b, c, Supplementary Fig. 26, and Supplementary Note 5), the fluxes of H_2_, CO_2_, O_2_, and N_2_ transporting from the feed to permeate chamber are 0.75, 0.0038, 0.0071, and 0.0063 molecule ns^−1^, respectively (each value are estimated from the average of four 200-ns-long MD simulations, except for the H_2_ flux) (Supplementary Table 5). The simulated selectivities of H_2_/CO_2_ (200) and H_2_/N_2_ (120) are comparable to their respective experimental values of 238 and 129 (Fig. 2b). From the mixed-gas separation simulations (Fig. 2d), the selectivities of H_2_/CO_2_ (162) and H_2_/N_2_ (90), averaged from four 300-ns-long MD simulations, are close to the corresponding experimental selectivities of 167 and 78 (Fig. 2b and Supplementary Table 6). Both the MD simulations and experiments show that the gas molecules with sizes much smaller than the free spacing between the neighboring nanosheets (e.g., H_2_ and He) move through the membrane quickly. By contrast, the gas molecules with sizes larger (or only slightly smaller) than the free spacing (O_2_, N_2_, and CH_4_) move 100 times slower because of the molecular sieving mechanism, resulting in gas separation selectivity above 100. For the gas molecule with specific adsorptive property, such as CO_2_, its interaction with MXene considerably affects the gas transport rate, which further increases the H_2_/CO_2_ selectivity. The quantitative agreement between the MD simulations and experiments indicates that molecular sieving occurs during gas separation through the MXene membrane. Generally, terminations on the surface of a 2D membrane may affect the separation performance in some cases, therefore, another model using –F termination (i.e., Ti_3_C_2_F_2_) has also been built to investigate the effect of different terminations on the MD simulated gas permeation (Supplementary Table 7, Supplementary Fig. 27, and Supplementary Note 6). The results show that there is no significant difference between the gas permeation in two simulation systems.

## Discussion

**Fig. 3:**
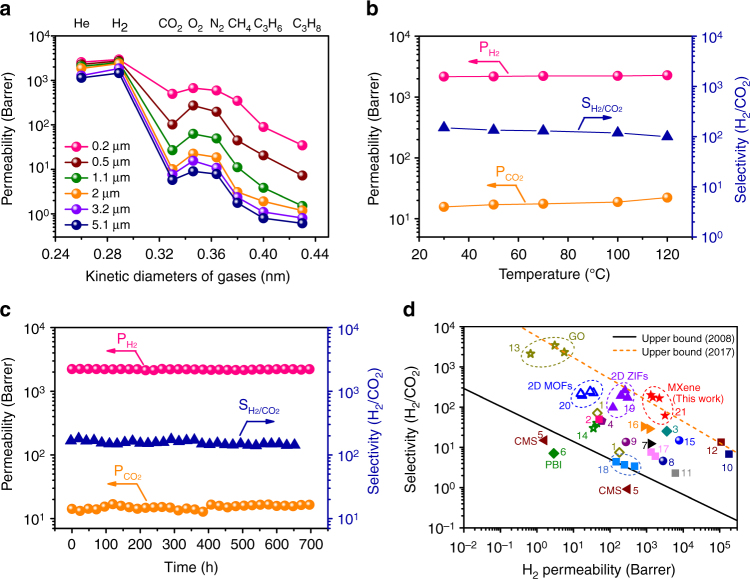
Gas separation performance of the MXene membrane. **a** Single-gas permeabilities through the MXene membranes with different thicknesses at 25 °C and 1 bar. **b** H_2_/CO_2_ separation performance of a 2-μm-thick MXene membrane as a function of temperature in the equimolar mixed-gas permeation. **c** Long-term separation of equimolar H_2_/CO_2_ mixture through a 2-μm-thick MXene membrane at 25 °C and 1 bar. **d** H_2_/CO_2_ separation performance of the MXene membrane compared with state-of-the-art gas separation membranes. The black line indicates the Robeson 2008 upper bound of polymeric membranes for H_2_/CO_2_ separation^58^, and the orange dashed line represents the 2017 upper bound of the best current membranes for H_2_/CO_2_ separation. Information on the data points is given in Supplementary Table 9

Moreover, the gas separation performance of the MXene membranes can be optimized by adjusting the membrane thickness, temperature, feeding H_2_ concentration, and feed gas pressure (Fig. 3a, b, Supplementary Figs. 28–33, and Supplementary Note 7). The MXene membrane shows stable performance during a 700 h continuous separation of H_2_/CO_2_ mixture (Fig. 3c). No deterioration was observed even when the feed gas contained 3 vol% steam (Supplementary Fig. 34). And the MXene membranes also show good reproducibility (Supplementary Table 8). Further, the 2-μm-thick MXene membrane also exhibits tensile strength above 50 MPa and Young’s modulus of 3.8 GPa, showing good mechanical properties (Supplementary Fig. 35 and Supplementary Note 8). Compared with various previously reported membranes (Fig. 3d, Supplementary Table 9, and Supplementary Note 9), the MXene membrane exhibits both great H_2_ permeability (>2200 Barrer) and high H_2_/CO_2_ selectivity (>160), which considerably exceeds the latest upper bound of most current membranes. This promising separation performance is attributed to the regular subnanometer channels in the stacked MXene membrane, and the pivotal role of the regular structure in separation have also been further verified using MD simulation (Supplementary Note 6).

The 2D structure and tunable physicochemical properties of MXene offer an exciting opportunity to develop a new class of molecular sieving membranes. Considering that more than 30 MXenes are already available^30^ and dozens more can be produced, there is certainly plenty of room for improving the performance even further. This work is significant for gas separation, such as H_2_ purification, e.g., in methanol reforming process, CO_2_ capture for zero-emission fossil fuel power generation, H_2_ recovery in ammonia production, etc. Furthermore, it also demonstrates a general concept for 2D membrane design with highly ordered nanochannels enabling fast and precise molecular sieving for mixture separation.

## Methods

### Preparation of the MXene membranes

The MXene solution was synthesized as follows^29^: one gram of LiF (purchased from Aladdin) was dissolved in 20 ml HCl (6 M, purchased from Sinopharm Chemical Reagent Co., Ltd.,) solution in a 250 ml Teflon beaker. Then, 1 g Ti_3_AlC_2_ (purchased from Beijing Jinhezhi Materials Co., Ltd.) was added to the solution with magnetic stirring at 35 °C for 24 h. The resulting product was washed using deionized (DI) water and centrifugated at 3500 rpm several times until the pH of the supernatant >6, and a clay-like sediment was obtained. The sediment was then dispersed in DI water with ultrasonication for 10 min in order to delaminate the MXene flakes. Most of the unexfoliated MXene was removed after centrifugation at 3500 rpm for 1 h. The concentration of the obtained MXene solution was ~0.5 mg ml^−1^. The MXene membranes were prepared by filtering a certain amount of the MXene solution on AAO (0.2 µm pore size and a diameter of 35 mm, purchased from Puyuannano Co., Ltd.) substrates using vacuum-assisted filtration (Supplementary Fig. 7). All membranes were dried at 70 °C for 24 h and could be easily detached from the substrate (Supplementary Fig. 8). During the membrane preparation process, Ar was used to prevent the oxidation.

### Characterization of the MXene nanosheets and membranes

SEM images were obtained using a Hitachi SU8220 device. The SEM elemental mapping analysis was conducted using an EDX (Oxford EDS, with INCA software). TEM images were obtained using a JEOL JEM-2100F microscope with an acceleration voltage of 200 kV. Elemental mapping in TEM was conducted using the Bruker EDS System. The XRD analysis was carried out using a Bruker D8 Advance with filtered Cu-Kα radiation (40 kV and 40 mA, *λ* = 0.154 nm); the step scan was 0.02°, the 2*θ* range was 2–10° or 2–70°, and the step time was 2 s. FTIR was conducted by Bruker VERTEX 33 units in the wavenumber range of 400–4000 cm^−1^. The XPS analysis was performed using an ESCALAB 250 spectrometer (Thermo Fisher Scientific) with monochromated Al-Kα radiation (1486.6 eV) under a pressure of 2 × 10^−9^ Torr. The AFM images were obtained using a Bruker Multi Mode 8 scanning probe microscope (SPM, VEECO) in tapping mode. The TG measurement was analyzed on a Netzsch STA 449F3 instrument under the flow of N_2_. The adsorption isotherms of H_2_, CO_2_, N_2_, and CH_4_ on the MXene membranes were measured using a Micromeritics (ASAP 2460) instrument. The mechanical tests were performed using an Instron-5565 universal testing machine (USA).

### Gas permeation measurements

All the gas permeation measurements were conducted by a homemade membrane module (Supplementary Fig. 20). Silicone gaskets were used to avoid the leakage and the direct contact between the stainless-steel module and membranes. The gas transport through the membrane was measured using the constant pressure, variable volume method. A calibrated gas chromatograph (GC, Agilent 7890A) was used to analyze the composition of the permeate gas. During single-gas permeation, a flow rate of 50 ml min^−1^ gas was used in the feed side of the membrane, and sweep gas with a flow rate of 50 ml min^−1^ was used to remove the permeated gas on the permeate side. During mixed-gas permeation, a gas mixture with a ratio of 1:1 was applied at the feed side of the MXene membrane, and the total flow rate of the feed gas was maintained at 100 ml min^−1^ (each gas at 50 ml min^−1^). The gas flow was controlled using mass flow controllers (MFCs). The pressures on both the feed and permeate side were maintained at 1 bar. In most cases, N_2_ was used as the sweep gas, except when using a N_2_-containing gas as the feed, then CH_4_ was employed as the sweep gas. The gas separation measurements were carried out at different temperatures. The membrane module was packed with heating tape and thermocouple and temperature controller devices were used to control the temperature and heating rate (2 °C min^−1^). Feed gases containing different H_2_ concentrations were obtained by adjusting the flow rates of H_2_ and CO_2_, which were controlled using the MFCs and calibrated using a bubble flowmeter. Steam (3 vol%) was introduced into the feed gas after passing it through a water tank at room temperature. The different gas pressures at the feed side of the MXene membranes were controlled with a back-pressure valve.

All of the gas permeation tests were carried out at least three times. The permeability of each gas was calculated from the following equation^6^:

1 $${\it{P}} = \frac{1}{{{\mathrm{\Delta }}{\it{p}}}} \times \frac{{273.15}}{{273.15 + {\it{T}}}} \times \frac{{{\it{P}}_{{\rm{atm}}}}}{{76}} \times \frac{{\it{L}}}{{\it{A}}} \times \frac{{{{\rm{d}}v}}}{{{{\rm{d}}{t}}}},$$

where *P* is the permeability (1 Barrer = 1 × 10^−10^ cm^3^ cm cm^−2^ s^−1^ cmHg^−1^ at standard temperature and pressure (STP)); Δ*p* is the transmembrane pressure (atm); *P*_atm_ is the atmospheric pressure (atm); *T* is the temperature (°C); *L* is the thickness of the membrane (cm); d*v/*d*t* is the volumetric displacement rate in the bubble flowmeter; and *A* is the effective area of the MXene membrane (1.13 cm^2^).

The selectivity of two components in the single-gas permeation (ideal selectivity) was calculated as follows:

2 $${\it{\alpha }} = \frac{{{\it{P}}_{\it{i}}}}{{{\it{P}}_{\it{j}}}},$$

where *P*_*i*_ and *P*_*j*_ are the permeability of each component.

The selectivity of two components in the mixed-gas permeation (separation factor) was calculated as follows:

3 $${\it{\alpha }}_{{\it{i}}{\mathrm{/}}{\it{j}}}{\it{ = }}\frac{{{\it{y}}_{\it{i}}{\mathrm{/}}{\it{y}}_{\it{j}}}}{{{\it{x}}_{\it{i}}{\mathrm{/}}{\it{x}}_{\it{j}}}},$$

where *x* and *y* are the volumetric fractions of the corresponding component in the feed and permeate side, respectively^7^.

### MD simulations

Classical MD simulations, which have been proven to be an efficient tool in similar studies^16,36,38^, were utilized to gain theoretical insight into the gas (e.g., H_2_, He, N_2_, O_2_, CO_2_, and CH_4_) permeation through the MXene membrane. Two sets of simulations were carried out: one to study the gas molecule permeation through the MXene membrane (Supplementary Fig. 26, denoted as the flux simulation)^16,36^, and another to calculate the gas diffusion coefficient in the MXene subnanometer channels (Supplementary Fig. 25, denoted as the confined diffusion simulation)^37,39^. In the flux simulation, 30 gas molecules were placed in the feed chamber on the left side of (along the *z* direction) the MXene membrane (Ti_3_C_2_O_2_) using a structure taken from the literature^40^. The free spacing between the MXene nanosheets was ~3.5 Å (see main text in Fig. 1h), and 2.4 wt% water as adsorbate was added randomly between the MXene nanosheets, as determined from the experiments (see the experimental TG in Supplementary Fig. 12). In order to investigate the effect of the surface functional groups on gas separation, gas permeation through the MXene membranes with another model (Ti_3_C_2_F_2_) has also been simulated and simulations of gas permeation were conducted through the MXene membrane again (H_2_ and CO_2_ as examples) (Supplementary Fig. 27 and Supplementary Table 7).

The MXene nanosheets were modeled by the UFF force field (FF) with QEq charge^41,42^, which has been proven to accurately simulate the interactions of gas molecules with nanoporous materials. The water was described using the SPC/E model^43^. The N_2_, O_2_, CO_2_, and CH_4_ gas molecules were modeled using the TraPPE FF^44,45^, and the united-atom parameters of H_2_ and He were taken from other publications^46,47^. These FF parameters have been proven to accurately simulate the transport of these five gases in nanoporous materials^39,48–50^. In both the flux or diffusion simulation, the system was subjected to a 500-step steepest-descent energy minimization. Then, a 200–300 ns (flux) or 40 ns (diffusion) NVT (constant particle number, volume and temperature) simulation was performed (leap-frog algorithm with a time step of 2 fs). The Nose–Hoover thermostat^51^ was employed to maintain a constant simulation temperature of 300 K. The MXene atoms were frozen in the simulations since the nanosheets were rather rigid. The short-range interactions were evaluated using a neighbor list of 10 Å that was updated every ten steps, and the Lennard–Jones interactions were switched off smoothly between 8 and 9 Å. A long-range analytical dispersion correction was applied to the energy to account for the truncation of these interactions^52^. The electrostatic interactions were evaluated using the reaction-field method^53^.

During the flux simulation, the gas molecules passed through the membrane to the permeate chamber (along the *z* direction), driven by the concentration difference, and the flux was calculated as the ratio of the number of gas molecules passing through the membrane to the simulation time. The MXene membrane was 5.5 nm × 5.3 nm in the primary simulation box, and a periodic boundary condition (PBC) was applied to the *x*–*y* direction (thus, the MXene membrane was essentially infinite in the *x–y* direction). The *z* length of the flux simulation box was 32 nm, and the MXene membrane (length ~5.3 nm) was placed approximately in the middle, leading to the feed chamber of ~12.6 nm long, and the permeate chamber of ~14.1 nm long.

Considering the lateral size of the MXene flakes in the experiments was 1–2 μm, confined diffusion simulations were also performed, in which six gas molecules (H_2_, He, N_2_, O_2_, CO_2_, and CH_4_) diffused between two MXene nanosheets (without the presence of a feed chamber or permeate chamber). These two MXene nanosheets with a free spacing of 0.35 nm were essentially infinite since the PBC was applied during the MD simulations, although they were 5.5 nm × 5.3 nm in the primary simulation box. During the confined diffusion simulation, the gas molecules diffused in two neighboring MXene nanosheets (a confined subnanochannel), and for each gas, a 40 ns NVT calculation was carried out and using the Einstein relation,

4 $${\it{D}} = \mathop {{{\rm{lim}}}}\limits_{{\it{t}} \to \infty } \frac{1}{{6{\it{t}}}}\left( {\frac{1}{{\it{N}}}\mathop {\sum}\limits_{\it{N}}^{{\it{k = }}1} {\left| {{\it{r}}_{\it{k}}({\it{t}}) - {\it{r}}_{\it{k}}(0)} \right|^2} } \right),$$

where *r*_*k*_(*t*) is the position of the *k*th molecule at time *t* and *N* is the number of molecules.

All MD simulations in this work were performed using the GROMACS 4.6.7 package^54,55^, while the simulation trajectories were analyzed using the GROMACS utilities and home-written codes. The interaction energies of the gas molecules with the MXene nanosheets were calculated from the diffusion simulation trajectories. Figures of the simulated systems were produced using VMD software^56^. Each flux simulation (200 ns for the single-gas permeation of H_2_, N_2_, O_2_, CO_2_ with 30 gas molecules in the simulation system; 300 ns for the mixed-gas permeation of H_2_ + N_2_ and H_2_ + CO_2_ with 60 gas molecules in the simulation system, 30 for each gas species) was repeated four times, and the averaged flux was reported. The flux simulations of the single-gas permeation (in which only one gas was used, e.g., H_2_, N_2_, O_2_, or CO_2_) were also performed with a very long permeate cell (~60 nm, denoted as the long-box simulations) to mimic the experiments more closely. The long-box simulations yielded very similar results compared to the normal-box simulation with a 14.1 nm long permeate cell (except for H_2_ in which the flux changed a little from 0.75 to 0.90 molecule ns^−1^). Thus, the flux simulation refers to the normal size box (*z* length of the box = 32 nm, permeate cell length = 12.6 nm, permeate cell length = 14.1 nm, main text Fig. 2d and Supplementary Fig. 26) in this work unless otherwise specified. Each diffusion simulation (H_2_, He, N_2_, O_2_, CO_2_, and CH_4_) was 40 ns long. Thus, the total simulation time was 5 μs or more (4 × 3 × 200 ns + 4 × 2 × 300 ns + 5 × 40 ns = 5 μs). See our previous publications for further simulation details^57^.

### Data availability

The data sets generated and analyzed during the current study are available from the corresponding authors on reasonable request.

## Electronic supplementary material

Supplementary Information

## References

[1] Sholl DS, Lively RP (2016). Seven chemical separations to change the world. Nature.

[2] Gin DL, Noble RD (2011). Designing the next generation of chemical separation membranes. Science.

[3] Park HB, Kamcev J, Robeson LM, Elimelech M, Freeman BD (2017). Maximizing the right stuff: the trade-off between membrane permeability and selectivity. Science.

[4] Liu G, Jin W, Xu N (2016). Two-dimensional-material membranes: a new family of high-performance separation membranes. Angew. Chem. Int. Ed..

[5] Gao J, Feng Y, Guo W, Jiang L (2017). Nanofluidics in two-dimensional layered materials: inspirations from nature. Chem. Soc. Rev..

[6] Kim HW (2013). Selective gas transport through few-layered graphene and graphene oxide membranes. Science.

[7] Shen J (2016). Subnanometer two-dimensional graphene oxide channels for ultrafast gas sieving. ACS Nano.

[8] Xu WL (2017). Self-assembly: a facile way of forming ultrathin, high-performance graphene oxide membranes for water purification. Nano Lett..

[9] Geim AK, Novoselov KS (2007). The rise of graphene. Nat. Mater..

[10] Koenig SP, Wang L, Pellegrino J, Bunch JS (2012). Selective molecular sieving through porous graphene. Nat. Nanotechnol..

[11] Wang L (2015). Molecular valves for controlling gas phase transport made from discrete ångström-sized pores in graphene. Nat. Nanotechnol..

[12] Abraham J (2017). Tunable sieving of ions using graphene oxide membranes. Nat. Nanotechnol..

[13] Chen L (2017). Ion sieving in graphene oxide membranes via cationic control of interlayer spacing. Nature.

[14] Tsapatsis M (2014). 2-Dimensional zeolites. AIChE J..

[15] Peng Y (2014). Metal-organic framework nanosheets as building blocks for molecular sieving membranes. Science.

[16] Wang X (2017). Reversed thermo-switchable molecular sieving membranes composed of two-dimensional metal-organic nanosheets for gas separation. Nat. Commun..

[17] Varoon K (2011). Dispersible exfoliated zeolite nanosheets and their application as a selective membrane. Science.

[18] Jeong HK, Nair S, Vogt T, Dickinson LC, Tsapatsis M (2003). A highly crystalline layered silicate with three-dimensionally microporous layers. Nat. Mater..

[19] Jeon MY (2017). Ultra-selective high-flux membranes from directly synthesized zeolite nanosheets. Nature.

[20] Celebi K (2014). Ultimate permeation across atomically thin porous graphene. Science.

[21] O’Hern SC (2014). Selective ionic transport through tunable subnanometer pores in single-layer graphene membranes. Nano Lett..

[22] Jain T (2015). Heterogeneous sub-continuum ionic transport in statistically isolated graphene nanopores. Nat. Nanotechnol..

[23] Tsou CH (2015). Effect of microstructure of graphene oxide fabricated through different self-assembly techniques on 1-butanol dehydration. J. Membr. Sci..

[24] Naguib M (2011). Two-dimensional nanocrystals produced by exfoliation of Ti_3_AlC_2_. Adv. Mater..

[25] Naguib M. in *Nanomaterials Handbook *2nd edn (Ed. Gogotsi, Y.) Ch. 4 (CRC Press, Boca Raton, 2017).

[26] Lukatskaya MR (2013). Cation intercalation and high volumetric capacitance of two-dimensional titanium carbide. Science.

[27] Ding L (2017). A two-dimensional lamellar membrane: MXene nanosheet stacks. Angew. Chem. Int. Ed..

[28] Shahzad F (2016). Electromagnetic interference shielding with 2D transition metal carbides (MXenes). Science.

[29] Alhabeb M (2017). Guidelines for synthesis and processing of two-dimensional titanium carbide (Ti_3_C_2_T_X_ MXene). Chem. Mater..

[30] Anasori B, Lukatskaya MR, Gogotsi Y (2017). 2D metal carbides and nitrides (MXenes) for energy storage. Nat. Rev. Mater..

[31] Magne D, Mauchamp V, Célérier S, Chartier P, Cabioc’h T (2016). Site-projected electronic structure of two-dimensional Ti_3_C_2_ MXene: the role of the surface functionalization groups. Phys. Chem. Chem. Phys..

[32] Lipatov A (2016). Effect of synthesis on quality, electronic properties and environmental stability of individual monolayer Ti_3_C_2_ MXene flakes. Adv. Electron. Mater..

[33] Halim J (2016). X-ray photoelectron spectroscopy of select multi-layered transition metal carbides (MXenes). Appl. Surf. Sci..

[34] Hope MA (2016). NMR reveals the surface functionalisation of Ti_3_C_2_ MXene. Phys. Chem. Chem. Phys..

[35] Li H (2013). Ultrathin, molecular-sieving graphene oxide membranes for selective hydrogen separation. Science.

[36] Joshi R (2014). Precise and ultrafast molecular sieving through graphene oxide membranes. Science.

[37] Sholl DS (2006). Understanding macroscopic diffusion of adsorbed molecules in crystalline nanoporous materials via atomistic simulations. Acc. Chem. Res..

[38] Nair RR, Wu HA, Jayaram PN, Grigorieva IV, Geim AK (2012). Unimpeded permeation of water through helium-leak-tight graphene-based membranes. Science.

[39] Verploegh RJ, Nair S, Sholl DS (2015). Temperature and loading-dependent diffusion of light hydrocarbons in ZIF-8 as predicted through fully flexible molecular simulations. J. Am. Chem. Soc..

[40] Khazaei M (2013). Novel electronic and magnetic properties of two-dimensional transition metal carbides and nitrides. Adv. Funct. Mater..

[41] Rappé AK, Casewit CJ, Colwell KS, Goddard WA, Skiff WM (1992). UFF, a full periodic table force field for molecular mechanics and molecular dynamics simulations. J. Am. Chem. Soc..

[42] Kadantsev ES, Boyd PG, Daff TD, Woo TK (2013). Fast and accurate electrostatics in metal organic frameworks with a robust charge equilibration parameterization for high-throughput virtual screening of gas adsorption. J. Phys. Chem. Lett..

[43] Berendsen H, Grigera J, Straatsma T (1987). The missing term in effective pair potentials. J. Phys. Chem..

[44] Potoff JJ, Siepmann JI (2001). Vapor-liquid equilibria of mixtures containing alkanes, carbon dioxide, and nitrogen. AIChE J..

[45] Martin MG, Siepmann JI (1998). Transferable potentials for phase equilibria. 1. United-atom description of n-alkanes. J. Phys. Chem. B.

[46] Darkrim F, Levesque D (1998). Monte Carlo simulations of hydrogen adsorption in single-walled carbon nanotubes. J. Chem. Phys..

[47] Talu O, Myers AL (2001). Molecular simulation of adsorption: Gibbs dividing surface and comparison with experiment. AIChE J..

[48] Rankin RB, Liu J, Kulkarni AD, Johnson JK (2009). Adsorption and diffusion of light gases in ZIF-68 and ZIF-70: a simulation study. J. Phys. Chem. C.

[49] Liu J, Keskin S, Sholl DS, Johnson JK (2011). Molecular simulations and theoretical predictions for adsorption and diffusion of CH_4_/H_2_ and CO_2_/CH_4_ mixtures in ZIFs. J. Phys. Chem. C.

[50] Wang H, Cao D (2015). Diffusion and separation of H_2_, CH_4_, CO_2_, and N_2_ in diamond-like frameworks. J. Phys. Chem. C.

[51] Feller SE, Zhang Y, Pastor RW, Brooks BR (1995). Constant pressure molecular dynamics simulation: the Langevin piston method. J. Chem. Phys..

[52] Shirts MR, Pitera JW, Swope WC, Pande VS (2003). Extremely precise free energy calculations of amino acid side chain analogs: comparison of common molecular mechanics force fields for proteins. J. Chem. Phys..

[53] Tironi IG, Sperb R, Smith PE, van Gunsteren WF (1995). A generalized reaction field method for molecular dynamics simulations. J. Chem. Phys..

[54] Hess B, Kutzner C, van der Spoel D, Lindahl E (2008). GROMACS 4: algorithms for highly efficient, load-balanced, and scalable molecular simulation. J. Chem. Theory Comput..

[55] Berendsen HJ, van der Spoel D, van Drunen R (1995). GROMACS: a message-passing parallel molecular dynamics implementation. Comput. Phys. Commun..

[56] Humphrey W, Dalke A, Schulten K (1996). VMD: visual molecular dynamics. J. Mol. Graph..

[57] Li L, Fennell CJ, Dill KA (2014). Field-SEA: a model for computing the solvation free energies of nonpolar, polar, and charged solutes in water. J. Phys. Chem. B.

[58] Robeson LM (2008). The upper bound revisited. J. Membr. Sci..

