# Supplementary material for: MXene molecular sieving membranes for highly efficient gas separation
Source: Nat Commun. 2018 Jan 11;9:155. doi: 10.1038/s41467-017-02529-6 (PMC5765169; doi:10.1038/s41467-017-02529-6)
Supplement: Supplementary file 1 — Supplementary Information [file 41467_2017_2529_MOESM1_ESM.pdf]

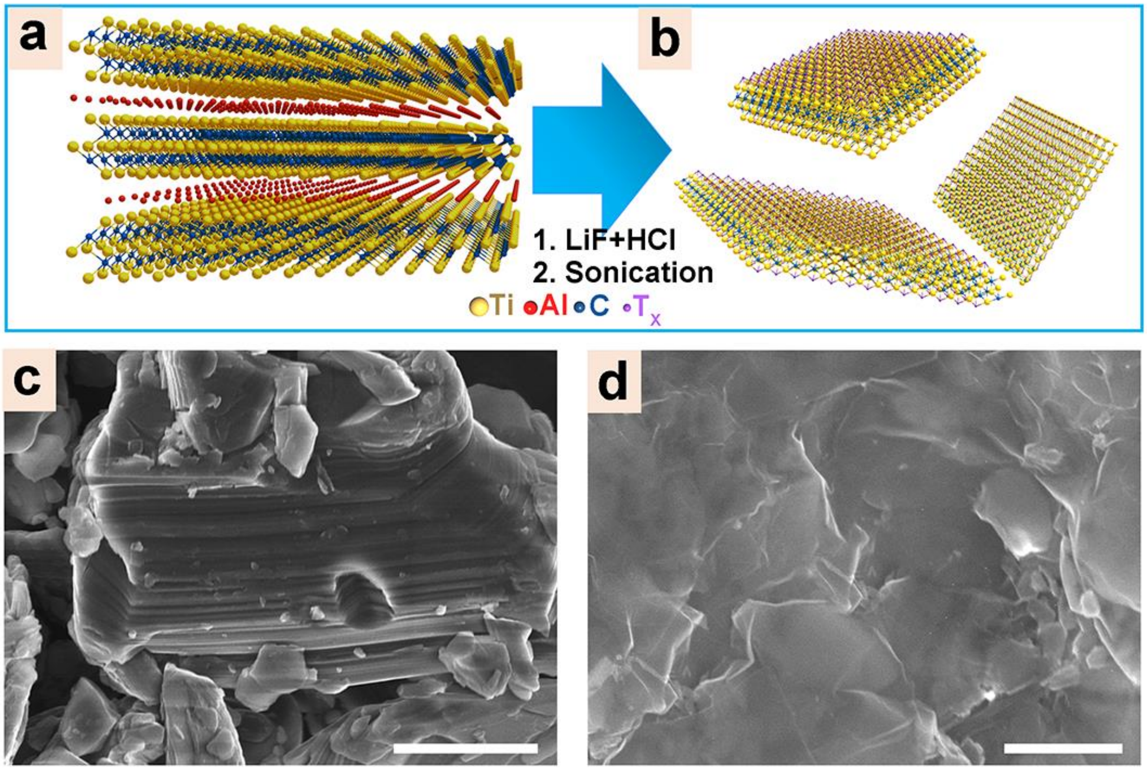


**Supplementary Figure 1. Morphological characterization of bulk MAX and exfoliated MXene nanosheets. a,** Architecture of the layered bulk phase of MAX. **b,** Illustration of the single-layer MXene nanosheets after etching A-layers and exfoliation. **c,** SEM image of bulk MAX precursor. **d,** SEM image of exfoliated MXene nanosheets. Scale bars: (c, d), 1 μm.

**Supplementary Note 1:** For a detailed explanation of the fabrication process, see Methods in the main text. $Ti_3AlC_2$ phase is a layered hexagonal structure with $P6_3/mmc$ symmetry, where Ti layers are nearly closed packed and the C atoms fill the octahedral sites[1]. For the MAX phase, layered solids connect each other by strong metallic, ionic and covalent bonds[1-4]. The $Ti_3C_2$ layers are interleaved with layers of Al atoms[5], thus, $Ti_3AlC_2$ phase structure can be described as 2D layers of $Ti_3C_2$ "glued" together with Al layers. The strong Ti-C bond has a mixed covalent/metallic/ionic character, whereas the Ti-Al bond is metallic[6]. Because the Ti-Al bonds are weaker than the Ti-C bonds in the MAX structure, the $Ti_3C_2T_X$ MXene nanosheets can be obtained by selected etching the Al layer of the MAX precursor using the mixture of HCl and LiF solution followed with a weak ultrasonication and centrifugation. The removal of the Al layers dramatically weakens the interactions between the $Ti_3C_2$ layers, thus allowing them to be readily separated[1]. In this process, the Al atoms are replaced by O, OH and/or F atoms, as the terminating groups on the MXene surface with different arrangements[1,7].

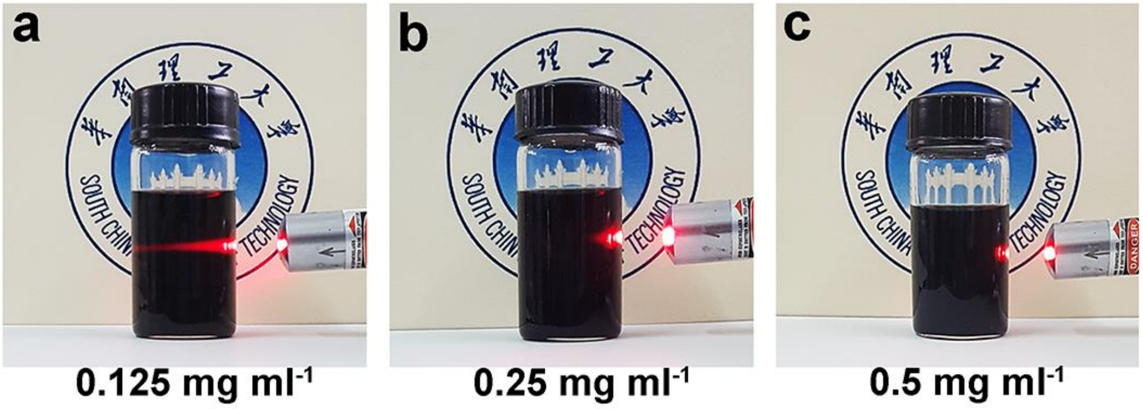


**Supplementary Figure 2. Tyndall scattering effect in the MXene colloidal suspension with different concentrations.** The Tyndall scattering effect in the colloidal suspension with the MXene concentration of 0.125 mg $ml^{-1}$ was obvious and the light beam went through the whole glass container, while the beam intensity became weaker with the increase of the MXene concentration.

**Supplementary Note 2:** The concentration of the colloidal solution was estimated by the gravimetric method. In detail, a blank AAO substrate was first weighed ($W_0$), then a certain amount of the solution ($V$) was filtered on it to form a supported membrane. Then the dried sample of the MXene membrane supported by AAO substrate was also weighed ($W$). Finally, the concentration of the colloidal solution ($C$) was be calculated by the formula as follows:

$$C = \frac{W - W_0}{V} \tag{1}$$

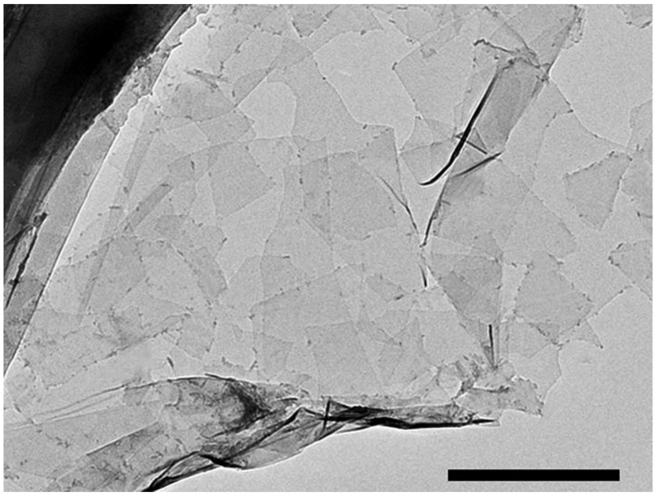

**Supplementary Figure 3. TEM image of the exfoliated MXene nanosheets.** Scale bar: 500 nm.

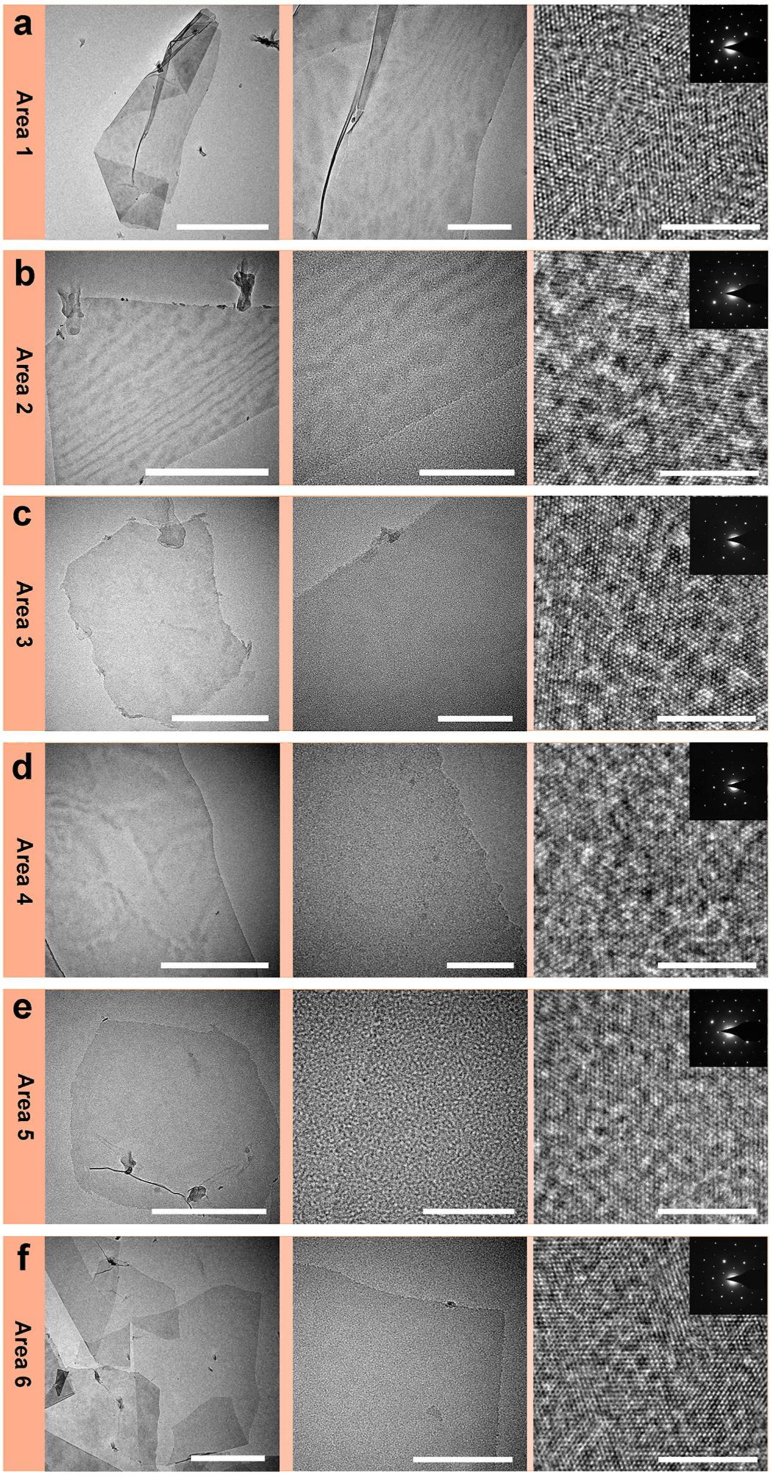


**Supplementary Figure 4.** TEM images and selected-area electron diffraction (SAED) patterns of MXene nanosheets in six randomly chosen areas. Scale bars: (a), 1 µm, 200 nm, 5 nm; (b), 500 nm, 100 nm, 5 nm; (c), 200 nm, 50 nm, 5 nm; (d), 500 nm, 20 nm, 5 nm; (e), 500 nm, 50 nm, 5 nm; (f), 1 µm, 200 nm, 5 nm; respectively.

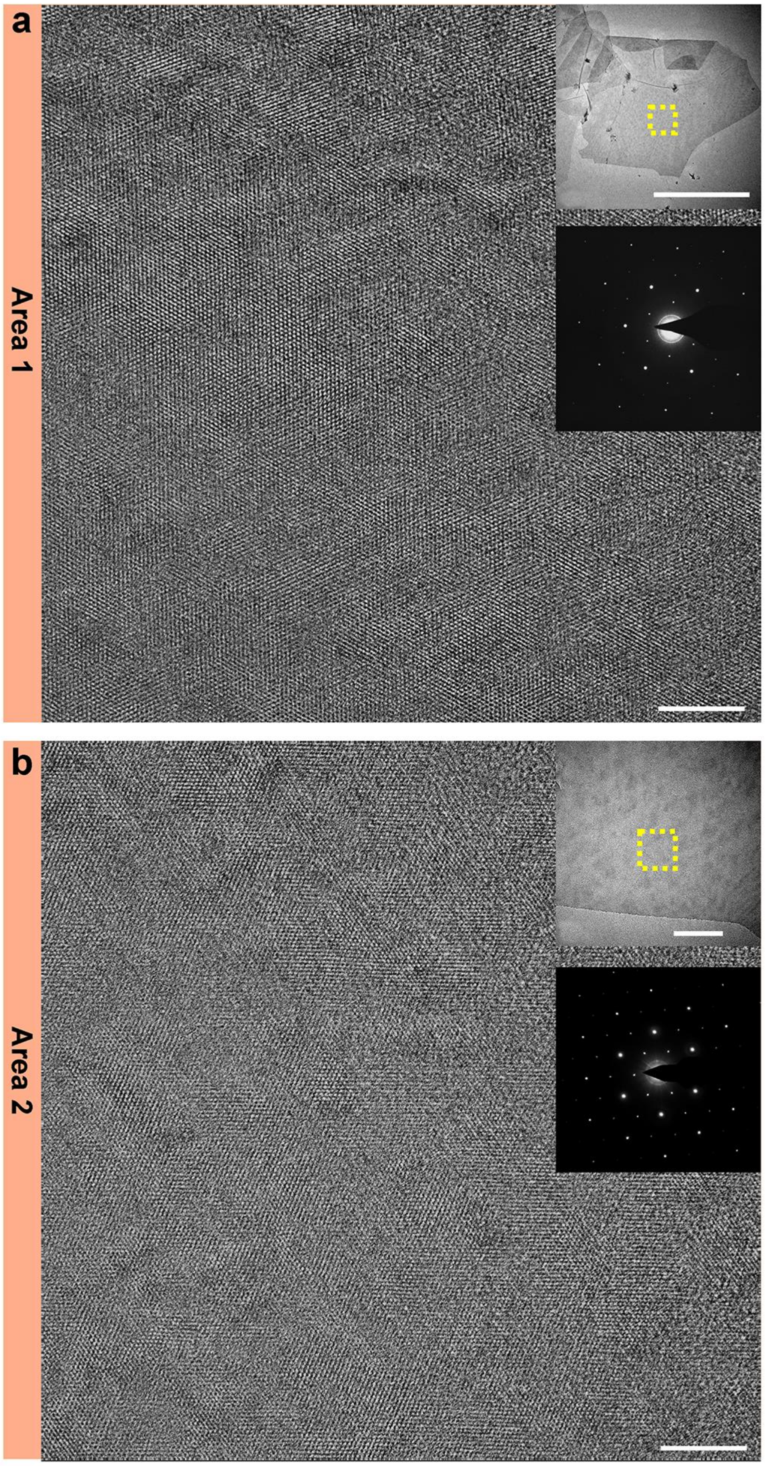


**Supplementary Figure 5.** Large-scale HRTEM images with randomly chosen area around 50 nm × 50 nm and selected-area electron diffraction (SAED) patterns of MXene nanosheets. Scale bars: (a, b), 5 nm; inset (a), 2 μm; inset (b), 200 nm.

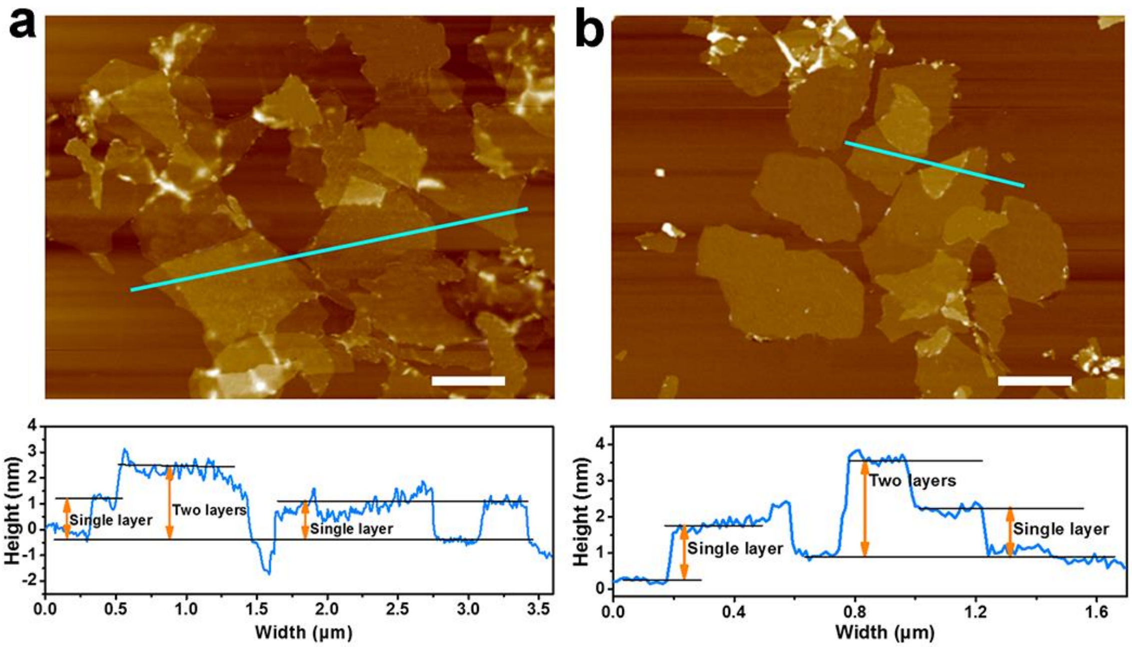


**Supplementary Figure 6. AFM images of the MXene nanosheets on fresh cleaved mica. The height profile of the nanosheets along the blue line is marked below. a,b,** Single layer with a thickness of approximately 1.5 nm, two layers have a total thickness of approximately 3 nm. The MXene nanosheets with a thickness of approximately 1.5 nm shown above are most likely single-layer flakes with some adsorbed water molecules and other impurities[8]. Scale bars: (a, b), 500 nm.

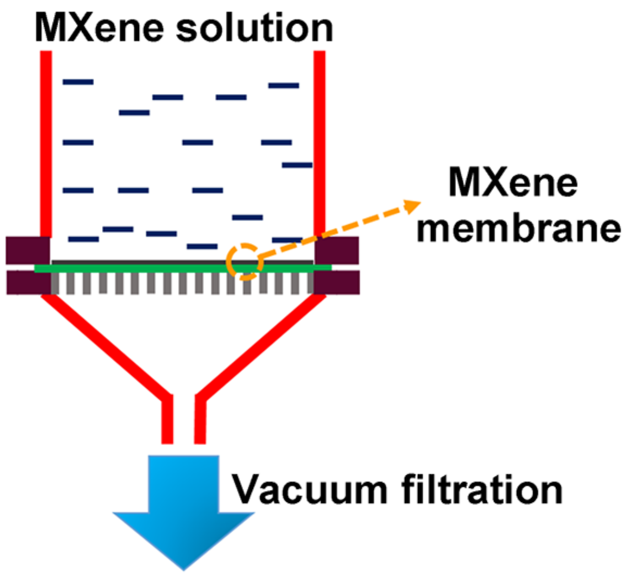


**Supplementary Figure 7. Preparation of a MXene membrane by simple vacuum assisted filtration.** The MXene colloidal suspension was filtrated on a porous AAO substrate with pore size of 200 nm under vacuum. In this process, MXene flakes construct the 2D layered MXene membrane.

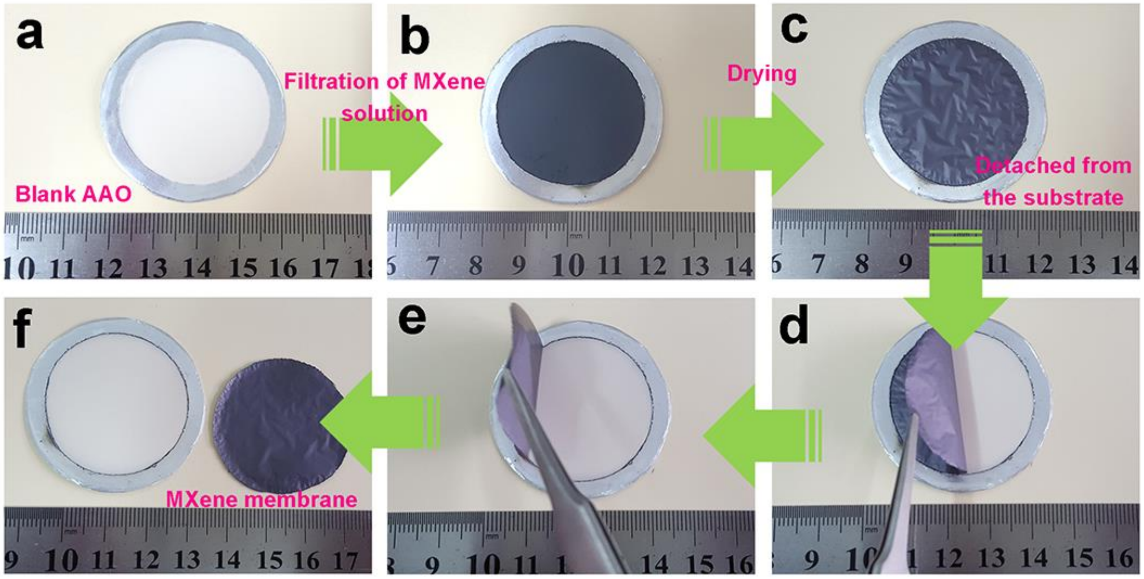


**Supplementary Figure 8. Free-standing MXene membrane after peeling from the porous AAO substrate. a,** Blank AAO substrate. **b,** A purple-black MXene membrane supported on AAO was obtained after filtration of the MXene suspension. **c,** After drying at 70 ℃ in vacuum oven for 24 h. **d,e,f,** The MXene membrane could be easily detached from the substrate producing a free-standing MXene membrane. The AAO substrate could be reused.

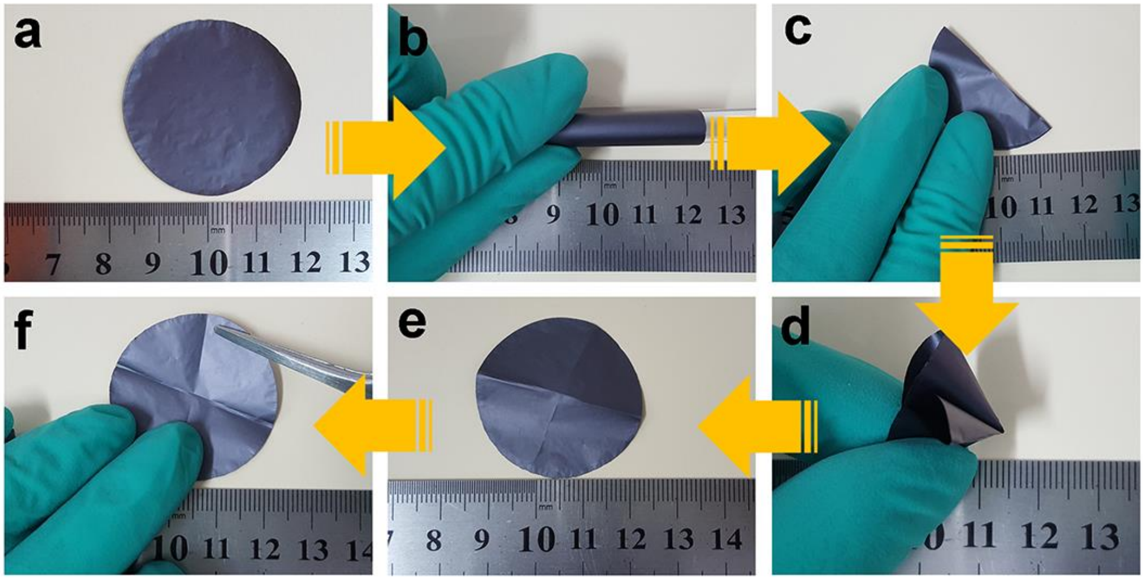


**Supplementary Figure 9. Good flexibility of the MXene membrane. a,** As-synthesized 2-μm-thick MXene membrane. **b-f,** It remained intact and undamaged even after rolling, folding and wrinkling.

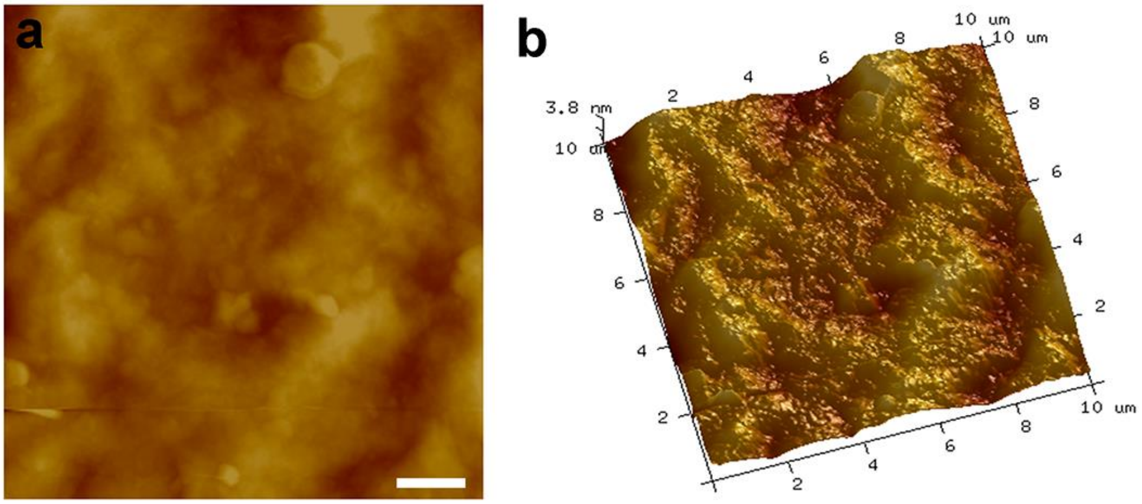


| | MXene membrane |
|---|---|
| **Rq (nm)** | 93.5 |
| **Ra (nm)** | 74.4 |

**Supplementary Figure 10. AFM images of the MXene membrane surface with a scan area of 10 μm × 10 μm. a,** 2D and **b,** 3D AFM images. The table below shows the surface roughness parameters of the MXene membrane. Scale bar: 1 μm.

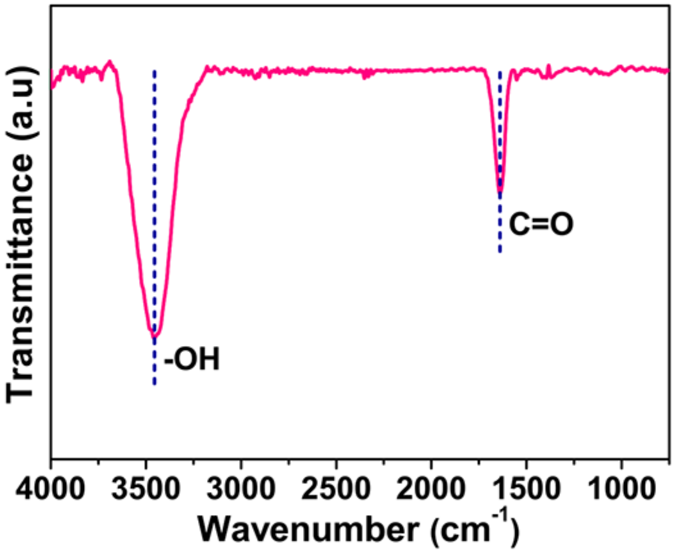


**Supplementary Figure 11. FTIR spectrum of the MXene membrane.** The stretching vibration at 3457 $cm^{-1}$ represents -OH, which demonstrates the -OH terminal groups on the MXene surface or the $H_2O$ molecules absorbed on it. The absorption band at 1641 $cm^{-1}$ corresponded to C=O from carbonyl or conjugated carbonyl groups, which might have resulted from the terminating groups connected to the edge of MXene nanosheets[2].

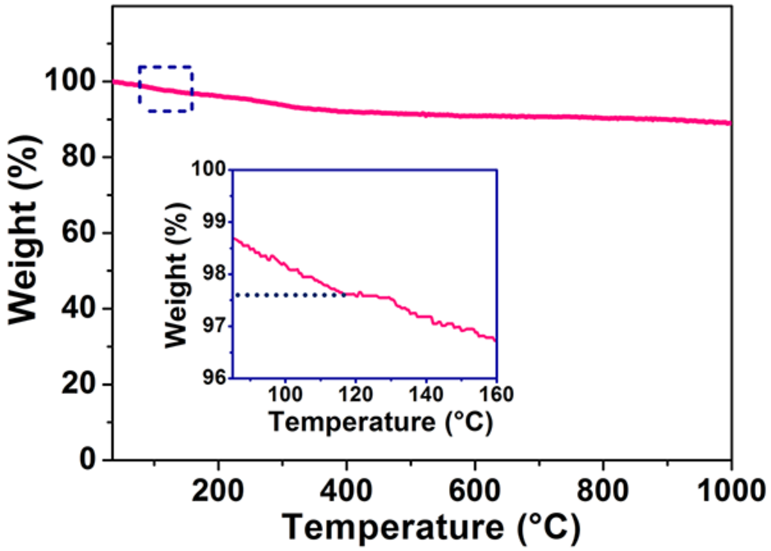


**Supplementary Figure 12. Thermogravimetric analysis result of the MXene membrane.** The curve shows a relatively good thermal stability of MXene membrane from room temperature to 1000 ℃ in nitrogen atmosphere with heating rate of 10 $^{o}$C min$^{-1}$, however there is a slight weight loss of the MXene membrane during heating process. The $H_2O$ content around 2.4 % could be deduced from the weight loss occurred at ~120 ℃[9], which is also in accordance with the XPS and FTIR results discussed before.

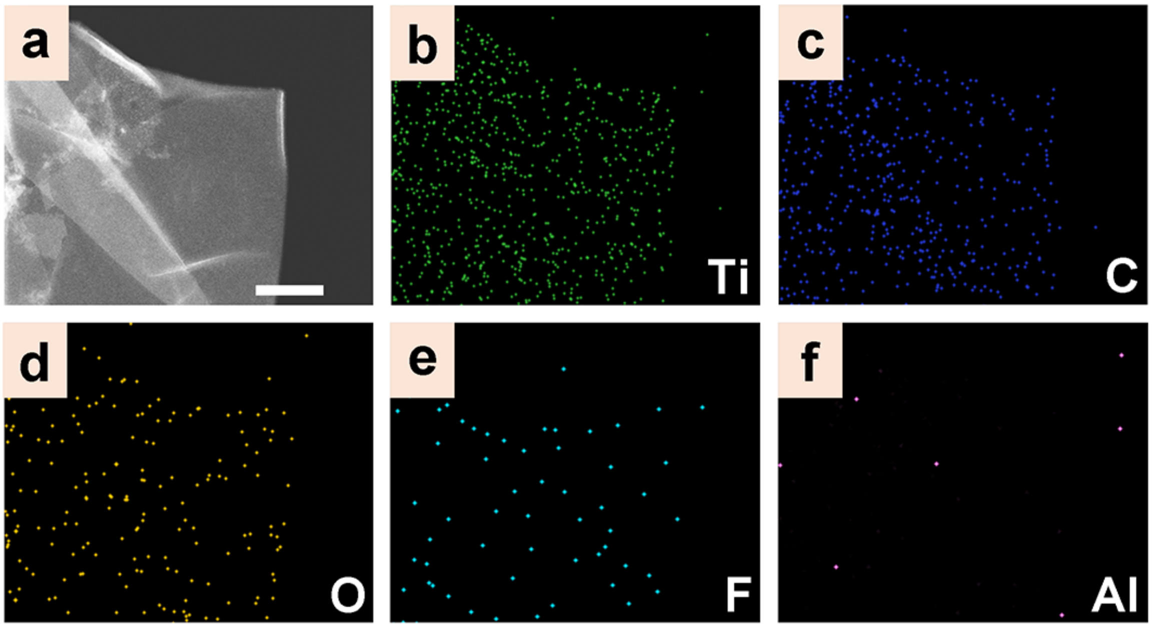


**Supplementary Figure 13. TEM image and the corresponding element distribution mappings of the MXene nanosheet.** Scale bar: 200 nm

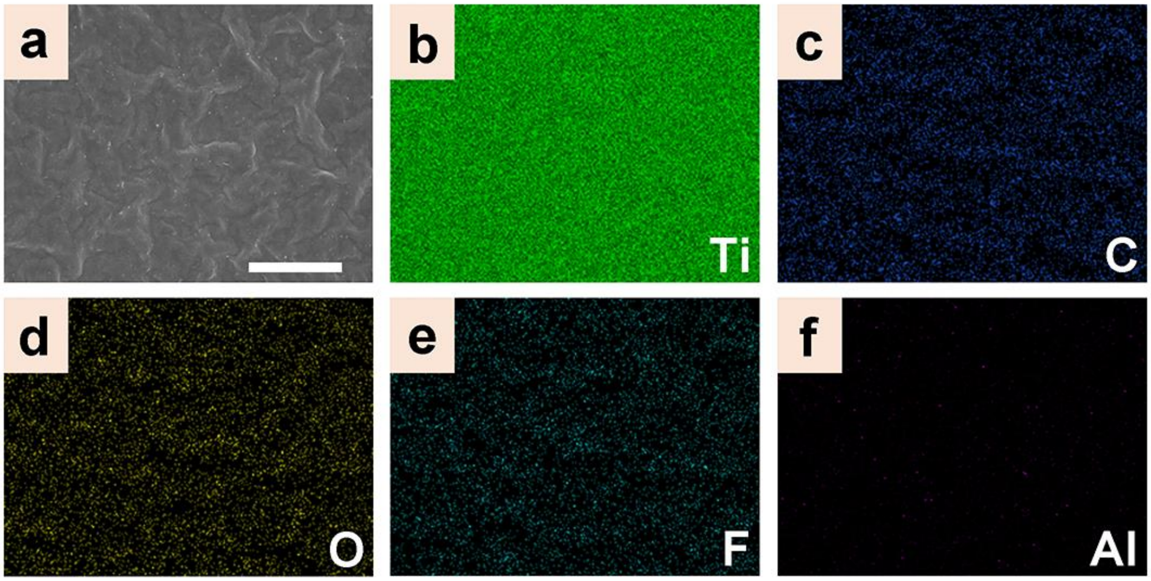


**Supplementary Figure 14. Top-view SEM image and the corresponding element distribution mappings of the MXene membrane.** Scale bar: 20 μm.

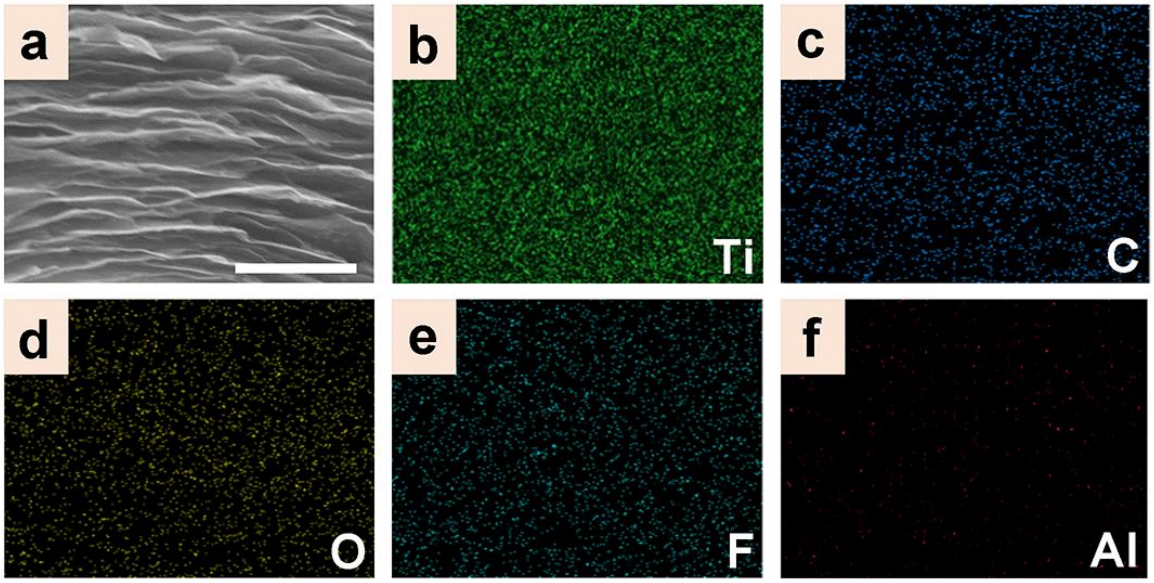


**Supplementary Figure 15. Cross-sectional SEM image and the corresponding element distribution mappings of the MXene membrane.** Scale bar: 1 μm.

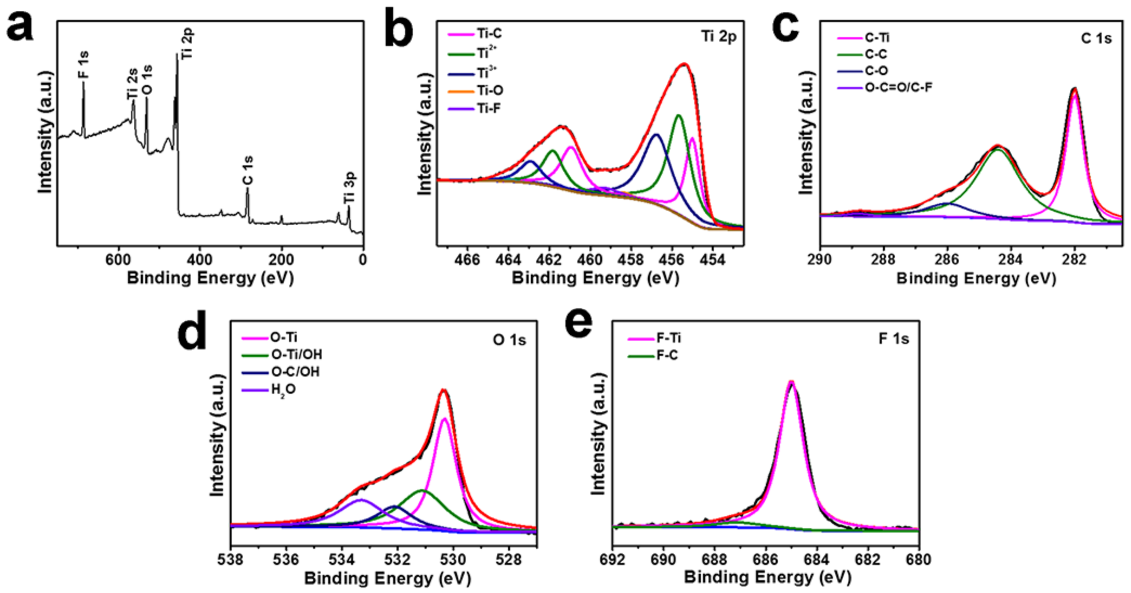


**Supplementary Figure 16. The XPS analysis presents the chemical states of the elements on the surface of the $Ti_3C_2T_X$ MXene membrane[10]. a,** The survey spectrum shows the presence of Ti, C, F and O. Four possible moieties of surface terminations existing in $Ti_3C_2T_X$ MXene, including C-Ti-$O_X$ (moiety I), C-Ti-$(OH)_X$ (moiety II), C-Ti-$F_X$ (moiety III) and $Ti_3C_2OH$-$H_2O$ ($H_2O$ads, moiety IV)[10]. **b,** In the Ti 2p region, the majority of the species are Ti atoms (Ti, $Ti^{2+}$, $Ti^{3+}$) that each belong to a mixture moieties of I, II, and/or IV, and the presence of Ti-F bond belongs to C-Ti-$F_X$ (viz. moiety III). The weak Ti-O bond shows the very low oxidation state of the membrane. The presence of the Ti-C and Ti-O bonds is evident from both spectra, indicating the formation of $Ti_3C_2T_X$ with oxygen-containing terminations after treatment. **c,** In the C1s region, the spectrum is fitted by three peaks. The largest peak is located at 282.0 eV binding energy corresponding to C-Ti-$T_X$ (moieties I, II, III, and/or IV). The other two peaks are assigned to graphitic C-C and C-O bounds. In addition, the weak peak at a high binding energy of 288.9 eV is assigned to the O-C=O and/or C-F groups[10-12]. **d,** In the O1s region, the fitted peaks are mainly assigned to C-Ti-$O_X$ (moiety I), C-Ti-$(OH)_X$ (moiety II) and $H_2O$ads (moiety IV). The $H_2O$ads component (moiety IV) reflects the presence of $H_2O$ in the MXene membrane. **e,** In the F1s region, two fitted peaks correspond to Ti-F and C-F bonds. Note that the presence of C-F is probably derived from the adsorption of exposed C after etching, and the O-C=O species are surface contaminations, similar to the result from the exposure of the high-surface area material to the ambient atmosphere[10-12].

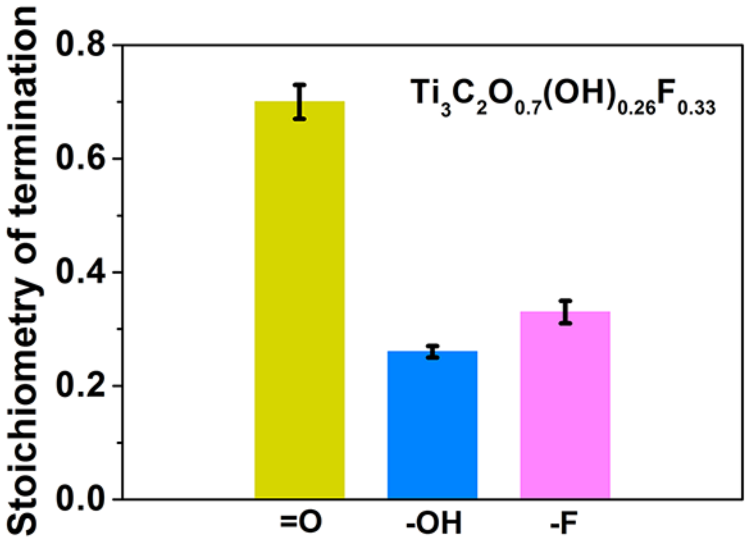


**Supplementary Figure 17.** Stoichiometric ratio of surface terminations in $Ti_3C_2T_X$ membrane, per $Ti_3C_2$ formula unit, assuming the charges of the oxygen atoms are −2, OH and F are −1. The equation is satisfied: $2n_O+n_{OH}+n_F=2$, yielding the following formulae[7,10], $Ti_3C_2O_{0.7}(OH)_{0.26}F_{0.33}$. Error bars derived from SD.

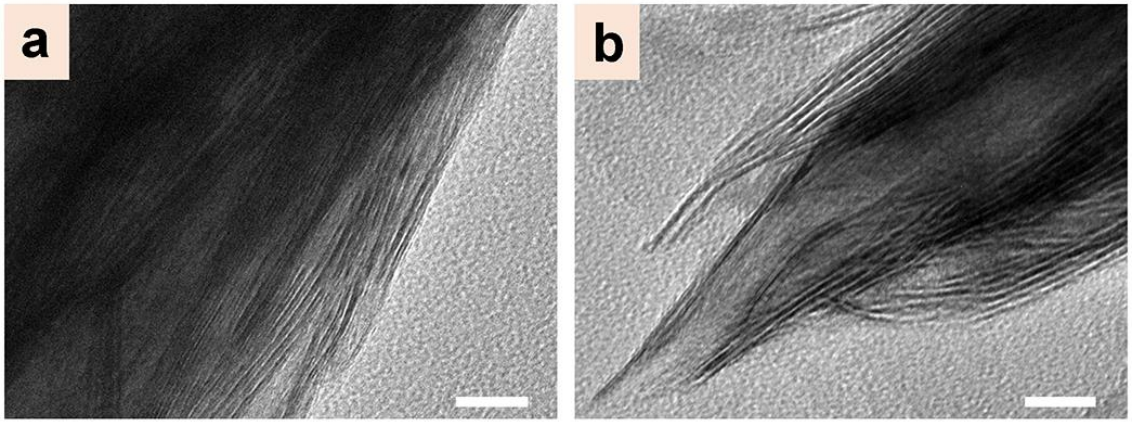


**Supplementary Figure 18. Cross-sectional TEM images of the MXene membrane with highly ordered 2D subnanometer channels.** Scale bars: (a, b), 10 nm.

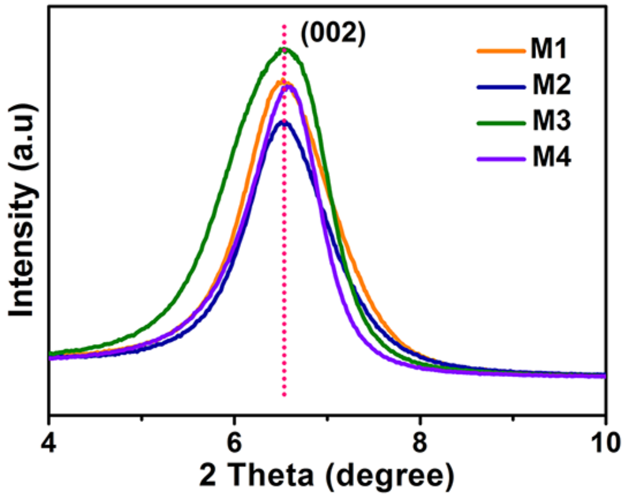


| MXene membrane | 2θ (degree) | *d*-spacing (Å) | Average *d*-spacing (Å) | Standard deviation of *d*-spacing |
|---|---|---|---|---|
| **M1** | 6.5236 | 13.55 | 13.53 | 0.063 |
| **M2** | 6.5498 | 13.50 | | |
| **M3** | 6.4952 | 13.61 | | |
| **M4** | 6.5645 | 13.47 | | |

**Supplementary Figure 19. XRD patterns of four MXene membranes at low 2θ angle.** The as-synthesized MXene membranes were characterized by XRD at low angles for *d*-spacing calculation. Four membranes were measured for increased accuracy. The table below shows the detailed parameters. The average *d*-spacing of the as-synthesized MXene membranes is 13.53 Å based on Bragg's Law with the standard deviation of 0.063, which shows reliable repeatability.

**Supplementary Note 3:** The crystalline structures of the MXene membranes were characterized by XRD; the *d*-spacing was calculated using Bragg's law:

$$n\lambda = 2d\sin\theta, \quad (2)$$

where $n$ is an integer (1, 2, 3…), $\lambda$ is the wavelength of the X-ray, $\theta$ is the incident angle and $d$ is the spacing between the diffraction planes [13].

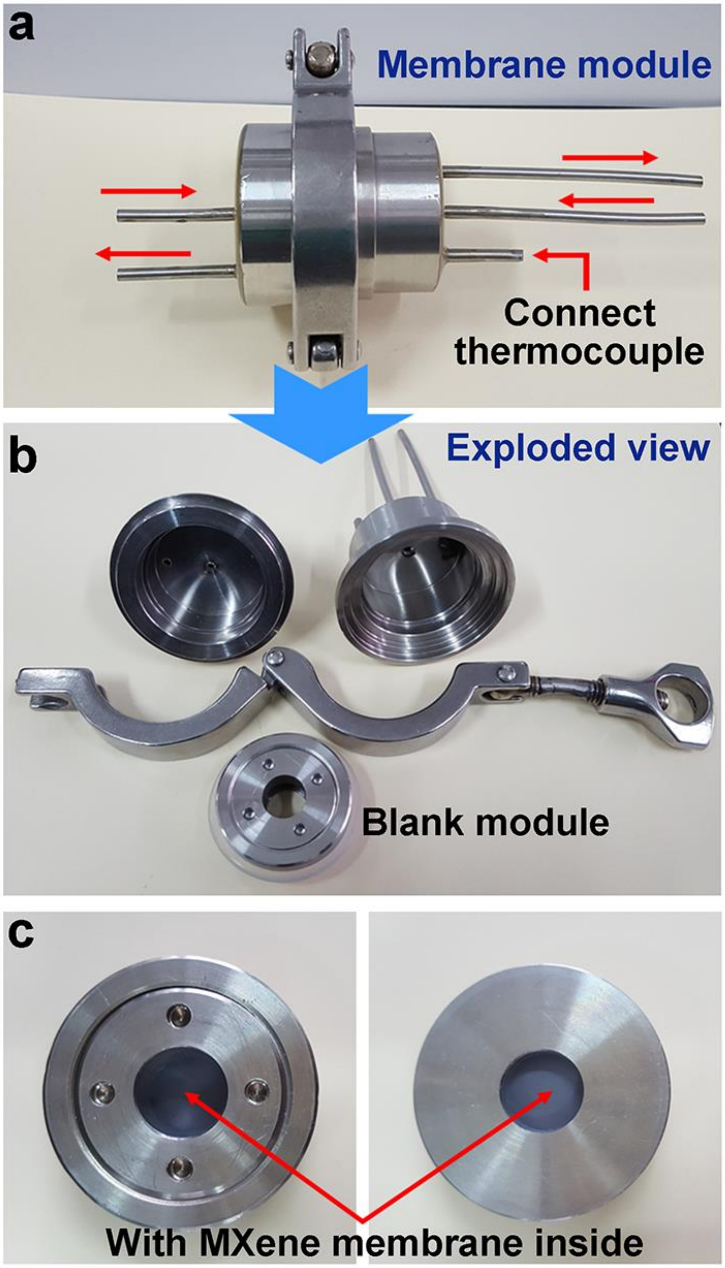


**Supplementary Figure 20. Photos of the membrane module for gas permeation. a,** Overview and **b,** inside view of the membrane module; **c,d,** top and down views of the cell within a MXene membrane.

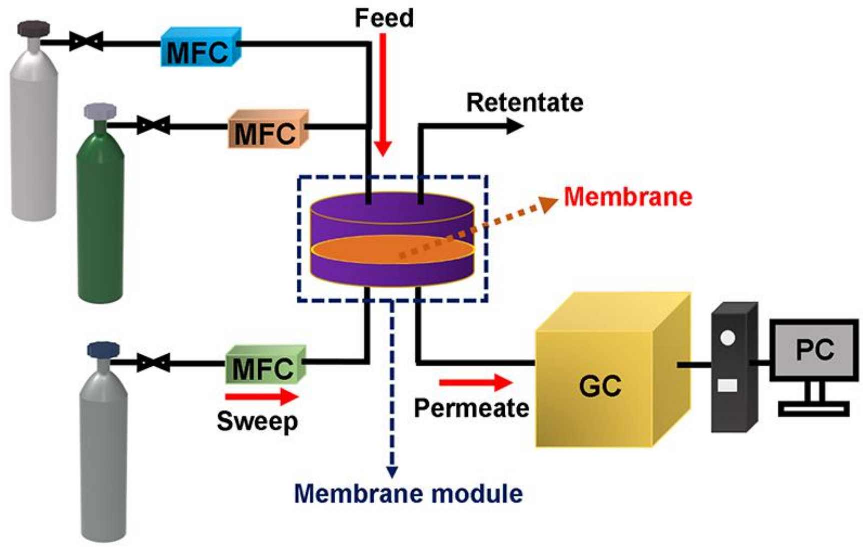


**Supplementary Figure 21. Apparatus scheme of a home-made Wicke-Kallenbach permeation cell for gas separation.** MFC: Mass flow controller (Qixinghuachuang, D07-19B). GC: Gas chromatograph (Agilent 7890A) with a thermal conductivity detector (TCD).

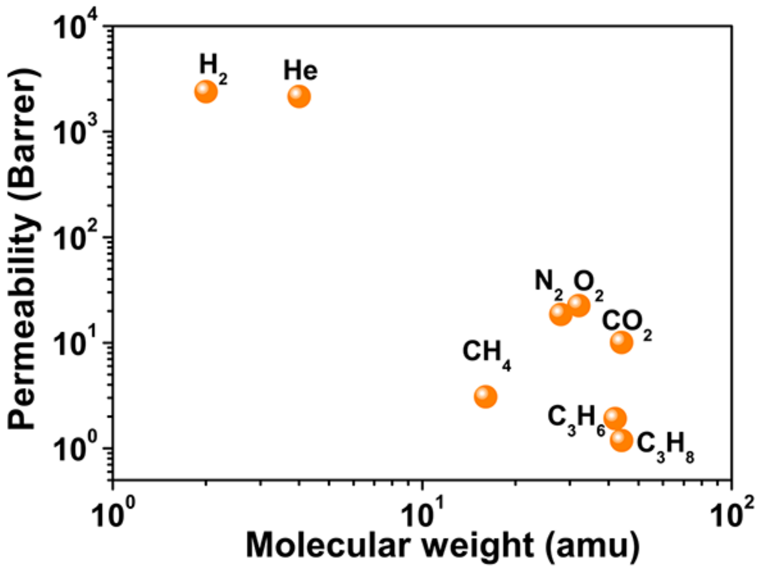


**Supplementary Figure 22. Single gas permeabilities through a 2-µm-thick MXene membrane as a function of gas molecular weight (amu, atomic mass unit) at 25 °C and 1 bar.** There is no obvious proportional relationship between the gas permeability and gas molecular weight.

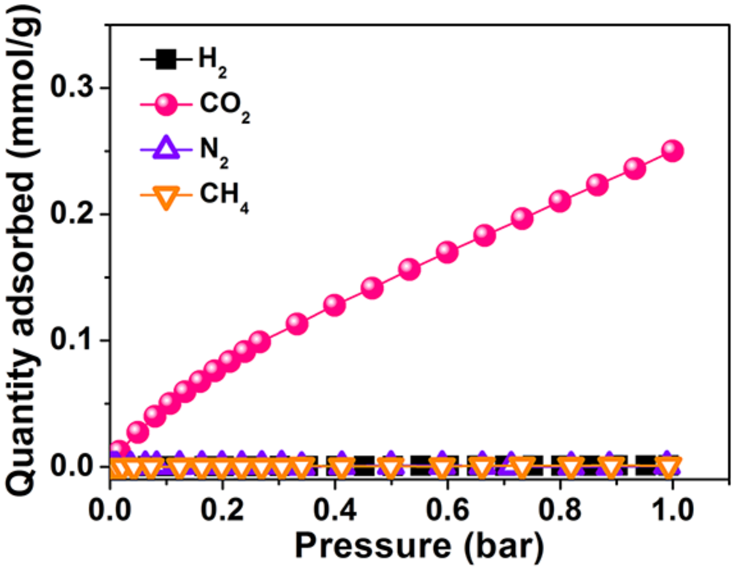


**Supplementary Figure 23. Adsorption isotherms of $H_2$, $CO_2$, $N_2$ and $CH_4$ on MXene membranes at 25 °C.**

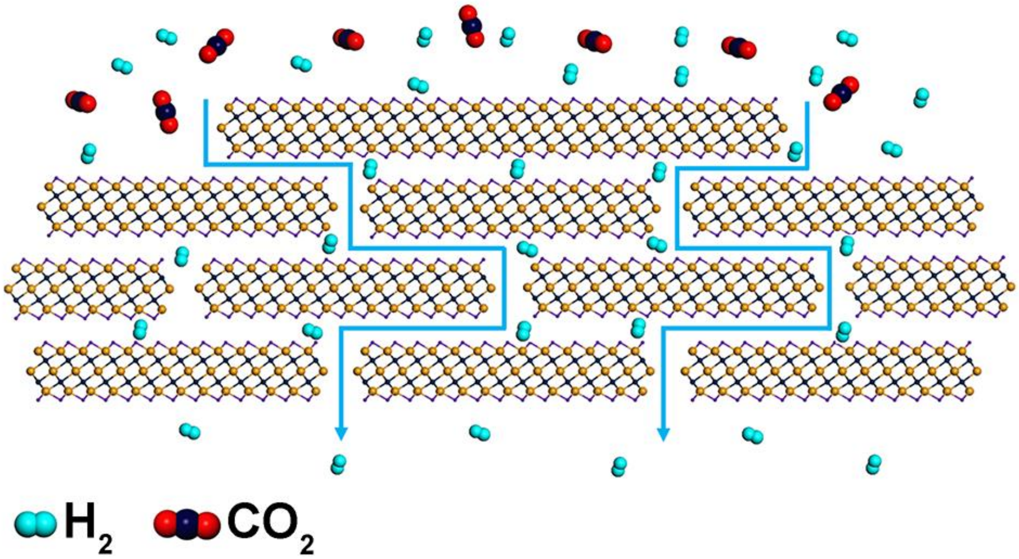


**Supplementary Figure 24. Molecular transport through stacks of nanosheets occurs in the interlayer spacing between neighboring nanosheets.** An example here is $H_2$ and $CO_2$ molecules passing through stacks of MXene nanosheets.

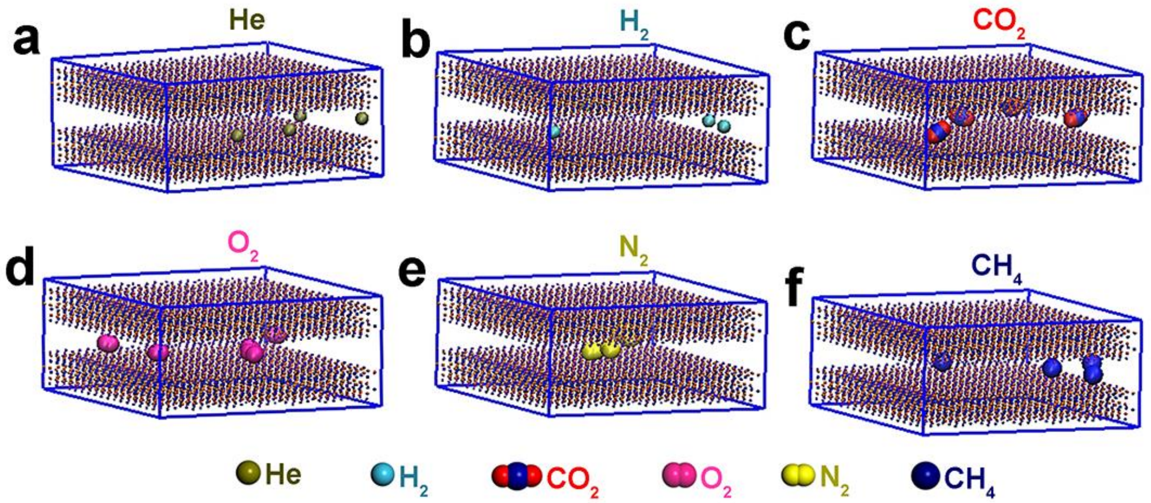


| Gas | He | $H_2$ | $CO_2$ | $O_2$ | $N_2$ | $CH_4$ |
|---|---|---|---|---|---|---|
| **Confined diffusion coefficient ($10^{-5}$ $cm^2$ $s^{-1}$)** | 9.45 | 12.858 | 0.0540 | 0.22 | 0.075 | 0.00605 |

**Supplementary Figure 25. Snapshots of the confined diffusion simulations, where gas molecules lied between two neighboring MXene nanosheets.**

**Supplementary Note 4:** Note $H_2$ and $CH_4$ were modeled by united-atom FF, where one particle represented one gas molecule. The table below shows the MD simulated gas confined diffusion coefficient in MXene nanosheets.

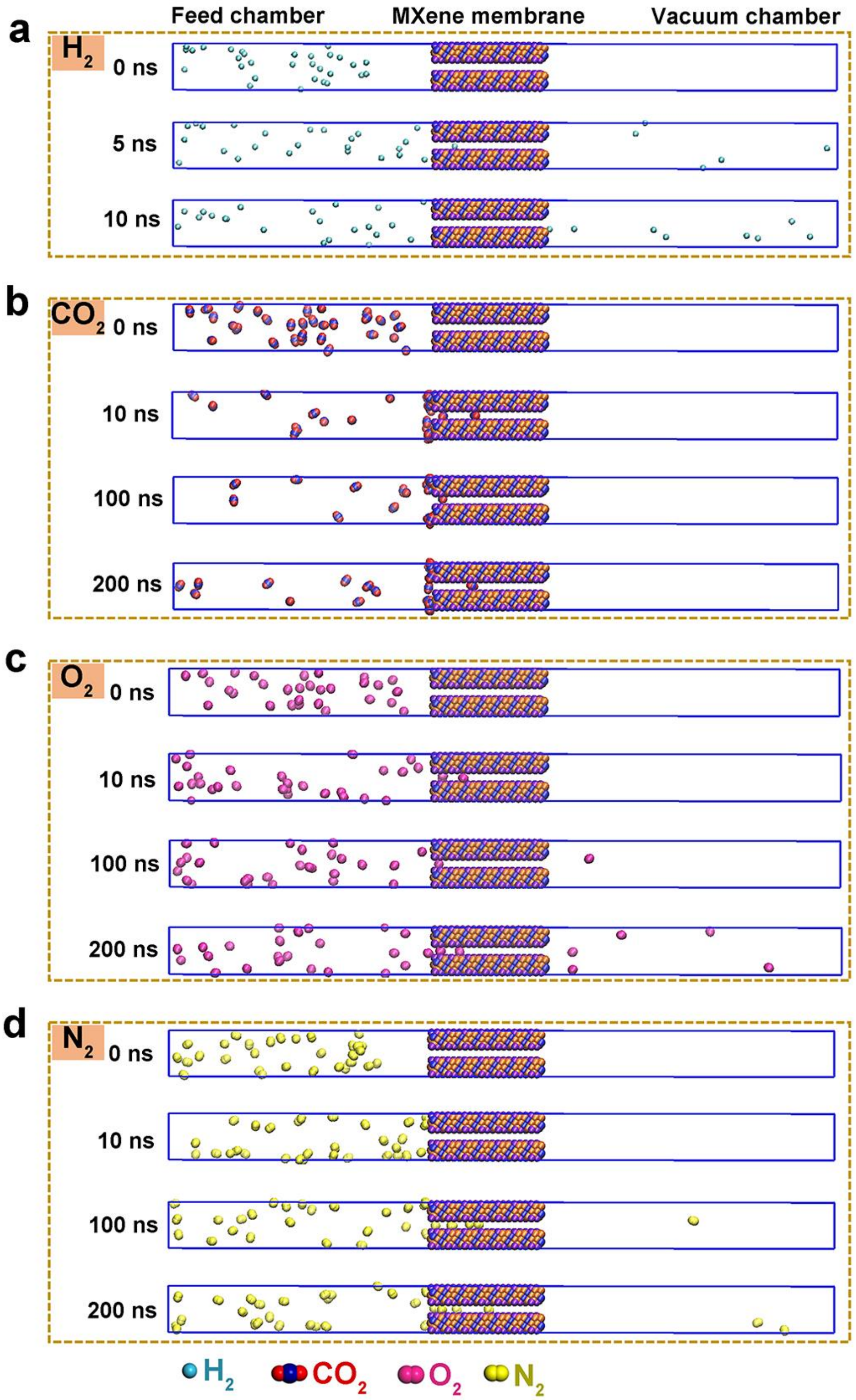


**Supplementary Figure 26. Snapshots of the single gas flux simulation at 0, 5, 10, 100 and 200 ns, including a, $H_2$, b, $CO_2$, c, $O_2$ and d, $N_2$.** The MXene membrane was in the middle of the simulation system, separating the feed (left) and permeate (right) chambers.

**Supplementary Note 5:** In the single gas flux simulation, the gas molecules diffused through the MXene membrane from the feed to the permeate chamber. Their fluxes are

different because of their different interactions with the MXene membrane. For $H_2$, only the first 10 ns of the simulation is shown because $H_2$ permeated through the MXene very quickly; approximately 8 $H_2$ molecules passed through the MXene membrane in 10 ns, while only 30 $H_2$ molecules in the entire simulation system. The equilibrium of $H_2$ permeation between the feed and permeate cell was reached quickly. Our MD simulations reveal that the $H_2$ permeated through the MXene membrane with a flux of 0.75 molecule $ns^{-1}$, as averaged from the first 10 ns of the four MD simulations. For $CO_2$, the first and second $CO_2$ molecules passed through the membrane at 110 ns and 160 ns, which indicated much slower gas permeation. For $O_2$, the first $O_2$ molecule passed through the membrane at 70 ns, and the second $O_2$ molecule passed through at 110 ns. For $N_2$, the first $N_2$ molecule passed through the membrane at 80 ns, and the second $N_2$ molecule passed through at 125 ns. The fluxes of $CO_2$, $O_2$ and $N_2$ were 0.0038, 0.0071 and 0.0063 molecule $ns^{-1}$; each was estimated from the average of four 200-ns-long NVT MD simulations (Supplementary Table 5). The $CO_2$ curve (red) in main text Fig. 2c fluctuates because $CO_2$ adsorption occurred on the MXene membrane, as indicated by the experimental isothermal adsorption (Supplementary Fig. 23). When the permeated $CO_2$ molecule re-adsorbed on the membrane, the number of $CO_2$ molecules in the permeate chamber decreased. When $CO_2$ desorbed from the membrane to the permeate chamber, the number of permeated $CO_2$ molecules increased to the former value. Therefore, the fluctuating $CO_2$ curve indicates the balance between the $CO_2$ adsorption and desorption on the MXene membrane. By contrast, the $N_2$ curve (orange) in main text Fig. 2c does not fluctuate, which agrees with the experimental finding that $N_2$ did not adsorb on the MXene. This result also agrees with the simulation results suggesting that $N_2$ had much weaker interactions with MXene (-97.5 kJ $mol^{-1}$) compared to $CO_2$ (-175.1 kJ $mol^{-1}$). In addition, using the $H_2$ flux (0.9) of the long-box simulation (feed chamber length = 12.6 nm, permeate chamber length = 60 nm, see the end of the MD simulation method section), the calculated selectivity of $H_2/CO_2$ and $H_2/N_2$ increased to 240 and 144, respectively.

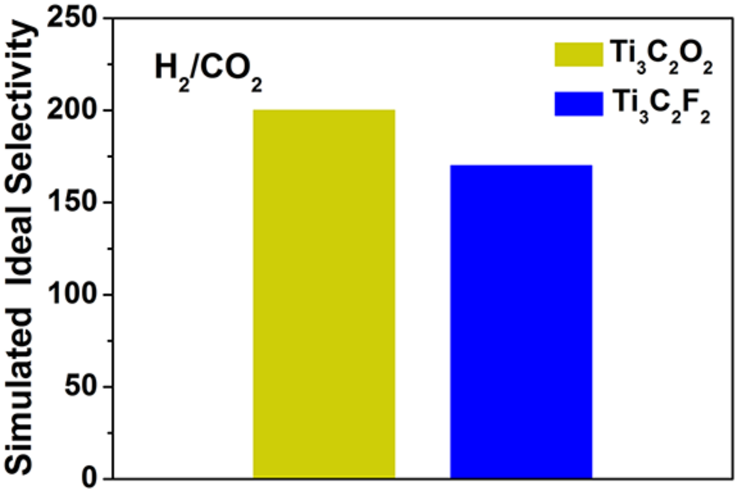


**Supplementary Figure 27. A comparison of simulated $H_2/CO_2$ selectivity with a different selection of MXene composition in molecular dynamics simulations.**

**Supplementary Note 6:** The terminal groups on the surface of 2D membrane may affect the separation performance to some extent. We have compared the separation performance of $Ti_3C_2O_2$ with $Ti_3C_2F_2$, but the difference is not significant (the ideal selectivity of $H_2/CO_2$ were 200 and 171 for $Ti_3C_2O_2$ and $Ti_3C_2F_2$, respectively), indicating that the effect of the terminating groups on the gas permeation performance is not significant. The insignificant difference may be attributed to the -O to -F transition not bringing sharp change to the MXene-gas molecule interactions.

The effect of the channel orderliness on the gas permeation has also been investigated by MD simulations. Taking into account that less orderliness would disturb the interlayer distance, and the uniform interlayer distance would be changed to non-uniform distance with a distribution, i.e. some interlayer distances are larger than 0.35 nm and some are smaller than 0.35 nm. Therefore, the confined diffusion coefficients of gas in the MXene nanochannels with different distances are simulated, demonstrating that gas molecules prefer to diffuse via wider channels with lower mass transfer resistance, which would dominate the gas separation performance. As a result, the membrane with wider interlayer distance gives much lower selectivity of $H_2/CO_2$ (e.g. membrane with interlayer distance of 0.45 nm gives a $H_2/CO_2$ selectivity of only ~70, which is much lower than the selectivity (> 200) with interlayer distance of 0.35 nm). In other words, deviations from 0.35 nm as little as 0.1 nm would deteriorate the gas selectivity significantly. Therefore, it can be indicated that our MXene membrane structure is highly-ordered, because slight disorder with only 0.1 nm disturbance to the interlayer distance would lead to a poor gas selectivity. Not to mention that disorder usually brings wide slits or flaws between MXene nanosheets, which would even ruin the gas selectivity.

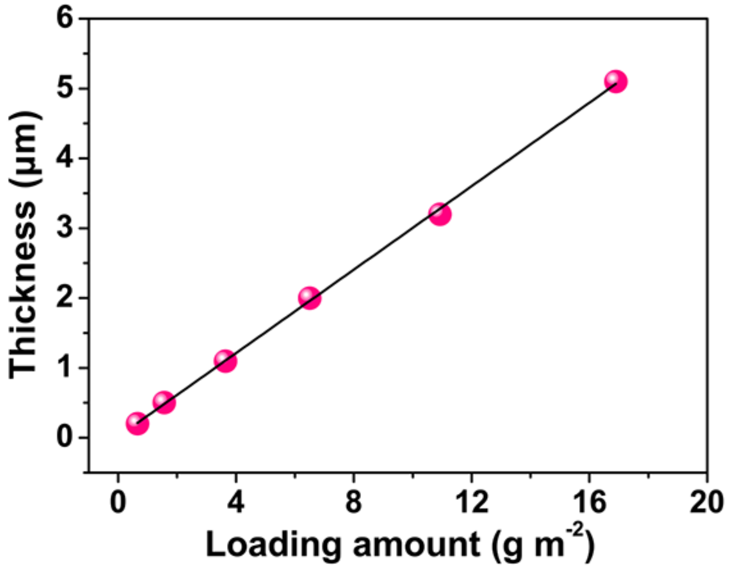


**Supplementary Figure 28. Relationship between the MXene membrane thickness and the loading amount of filtrated MXene solution.** MXene membranes with thicknesses of 200 nm, 500 nm, 1.1 μm, 2.0 μm, 3.2 μm and 5.1 μm could be obtained by adjusting the loading amount of filtered MXene solution. The corresponding cross-sectional SEM images of the MXene membranes are shown in Supplementary Fig. 29.

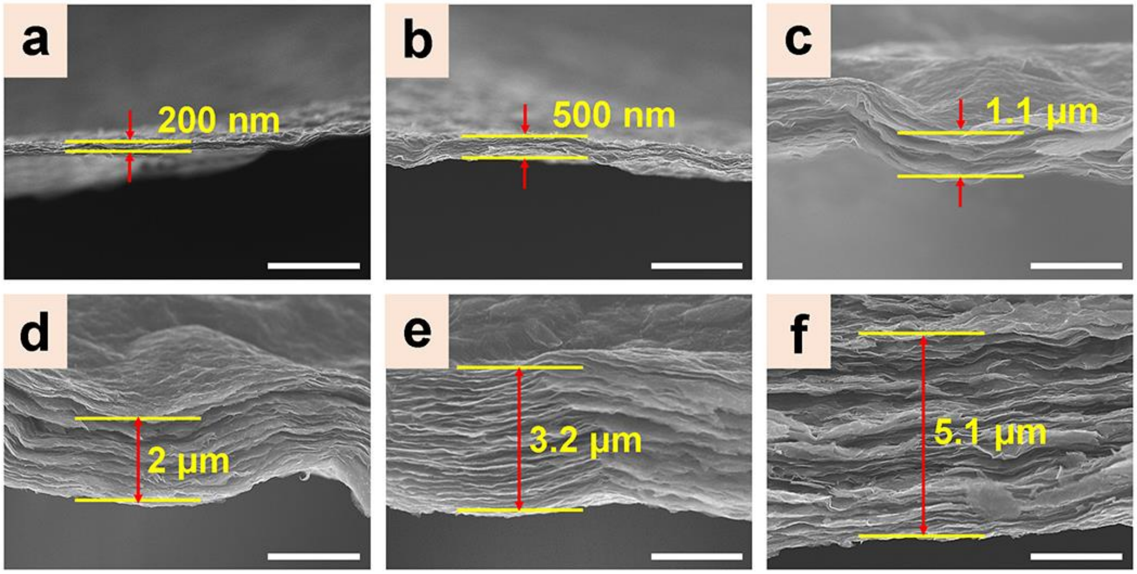


**Supplementary Figure 29. Cross-sectional SEM images of the MXene membranes with different thicknesses from 0.2 μm to 5.1 μm.** Scale bars: (a-f), 2 μm.

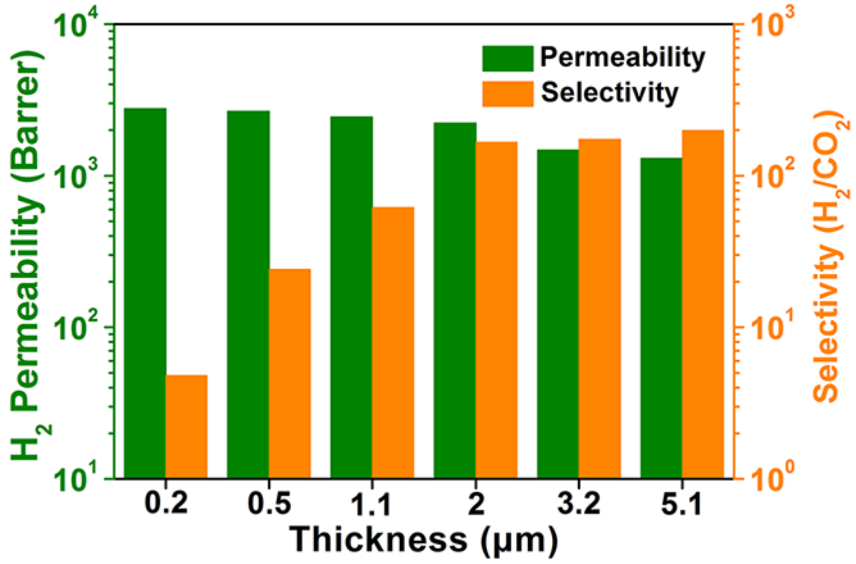


**Supplementary Figure 30. $H_2$ permeability and $H_2/CO_2$ selectivity for series MXene membranes as a function of membrane thickness.** Sharp cut-off of the permeability between small gases (He and $H_2$) and larger ones for each membrane are apparent (Main text Fig. 3a). The gas permeability decreases with increasing membrane thickness due to the prolonged gas diffusion pathway, while the selectivity increases.

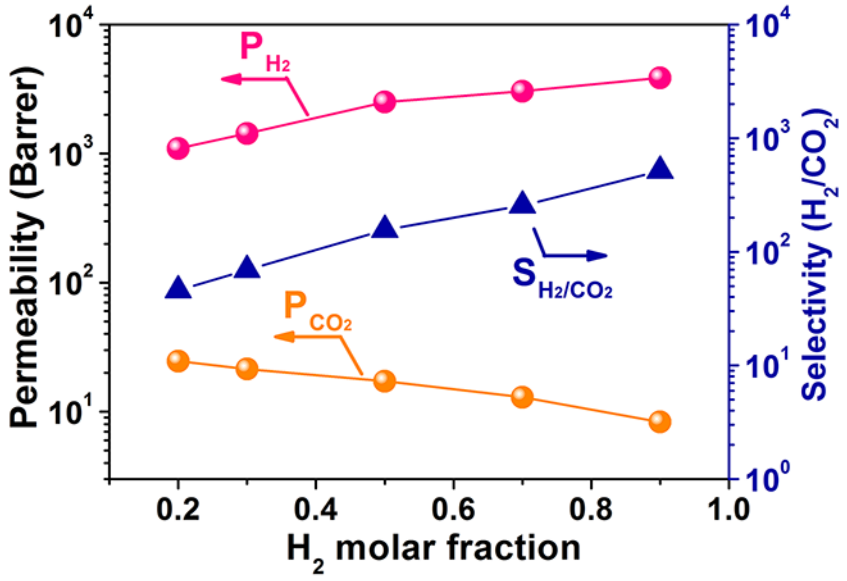


**Supplementary Figure 31. Effect of $H_2$ concentration in the feed gas on the separation performance of a 2-μm-thick MXene membrane with mixed $H_2/CO_2$ gas feeding.** The $H_2$ permeability increases with the increasing $H_2$ concentration in the feed gas due to the enhanced driving force for gas permeation through the MXene membrane, while $CO_2$ permeability decreases. As a result, the $H_2/CO_2$ selectivity jumps up to ~600 when feeding with gas mixture of (90% $H_2$ + 10% $CO_2$).

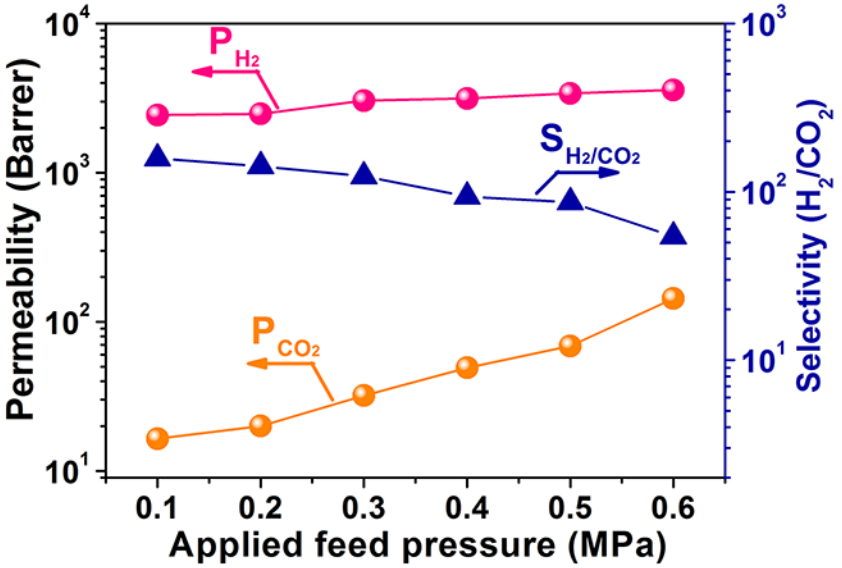


**Supplementary Figure 32.** **$H_2/CO_2$ separation performance of a 2-μm-thick MXene membrane as a function of the gas pressure in the feed side with equimolar mixed gas feeding.**

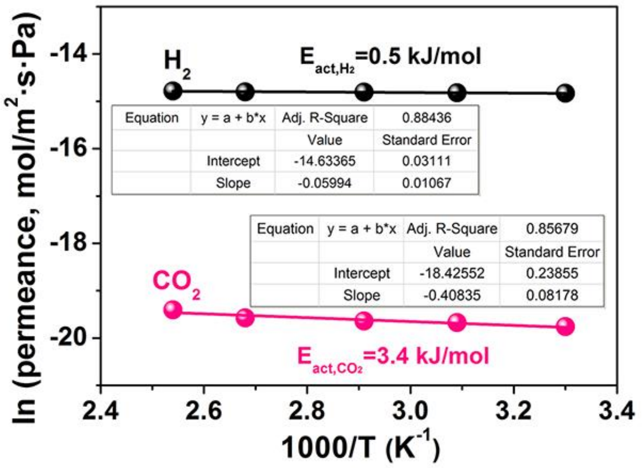


**Supplementary Figure 33. Arrhenius temperature dependence of $H_2$ and $CO_2$ permeance through the MXene membrane at 25 °C with equimolar mixed gas feeding.**

**Supplementary Note 7:** The temperature dependence of gas permeation can be stated by the Arrhenius equation:

$$P = Aexp(-\frac{E_{act}}{RT}) \tag{3}$$

$$lnP = -\frac{E_{act}}{R} \cdot \frac{1}{T} + C \tag{4}$$

where $P$ is the gas permeance, $A$ is the pre-exponential factor, $E_{act}$ is the apparent activation energy, $R$ is the ideal gas constant (8.314 J mol$^{-1}$ K$^{-1}$) and $T$ is the absolute Kelvin temperature (K). $ln(P)$ versus $1/T$ displays a straight line, whose slope is used to calculate $E_{act}$. As shown in Supplementary Fig. 33, the $E_{act,H_2}$ is about 0.5 kJ mol$^{-1}$, and $E_{act,CO_2}$ is about 3.4 kJ mol$^{-1}$.

The apparent activation energy is an association of diffusion activation energy and heat of adsorption,

$$E_{act} = E_{diff} - \Delta H_{ads} \tag{5}$$

considering much weaker adsorption of $H_2$ on MXene than $CO_2$, heat of adsorption of $CO_2$ on MXene is also expected to be higher than $H_2$. Therefore, diffusion activation energy of $CO_2$ through the MXene membrane is at least 3.4 kJ mol$^{-1}$ higher than that of $H_2$, which indicates much more activated diffusion of $CO_2$ through MXene membranes or much tighter fit of $CO_2$ with MXene flakes. It can also explain why the $H_2/CO_2$ separation factor decreases with increasing temperature (Main text Fig. 3b). Because $CO_2$ permeability rises faster than that of $H_2$, there is more activated $CO_2$ diffusion than $H_2$ in the MXene membrane.

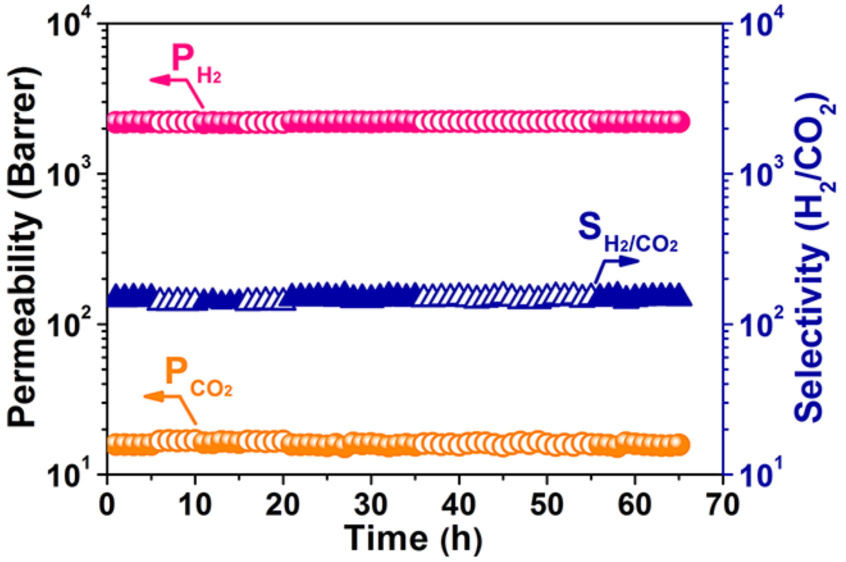


| Feeding gas | Permeability (Barrer) | | Selectivity ($H_2/CO_2$) |
|---|---|---|---|
| | $H_2$ | $CO_2$ | |
| **Dry** | 2214.7 | 15.8 | 149 |
| **Wet** | 2208.3 | 16.7 | 140 |
| **Dry** | 2186.0 | 16.3 | 142 |
| **Wet** | 2195.2 | 16.6 | 140 |
| **Dry** | 2221.8 | 15.7 | 150 |
| **Wet** | 2228.1 | 15.9 | 148 |
| **Dry** | 2216.9 | 15.7 | 149 |

**Supplementary Figure 34. $H_2/CO_2$ separation performance of a 2-µm-thick MXene membrane as a function of time with shifting between dry and wet (add 3 vol % steam) equimolar mixed gas feeding.** Solid symbol: dry feeding; open symbol: wet feeding. The table below shows the detailed permeability and selectivity.

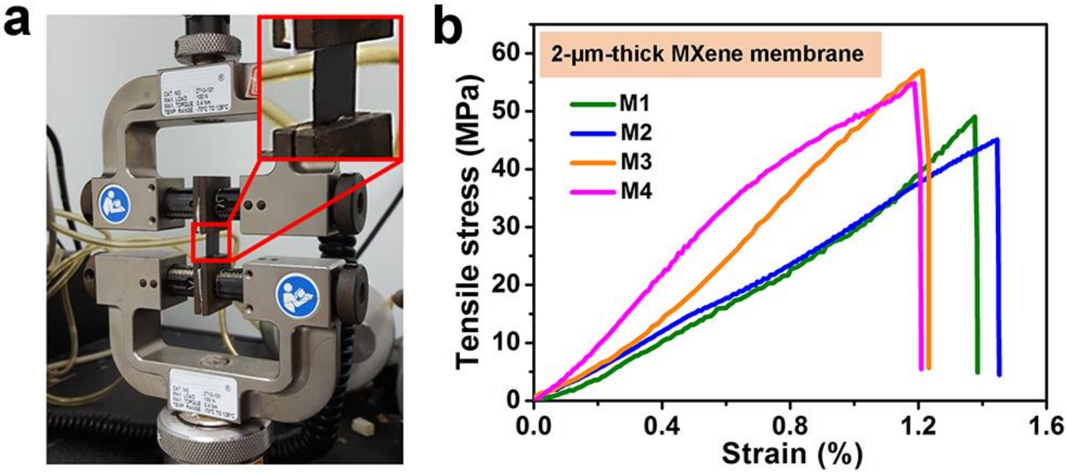


| MXene membrane | Tensile strength (MPa) | Young's modulus (GPa) | Strain to failure (%) |
|---|---|---|---|
| **M1** | 49.0 | 3.75 | 1.4 |
| **M2** | 45.1 | 3.62 | 1.5 |
| **M3** | 57.0 | 3.90 | 1.2 |
| **M4** | 54.8 | 3.94 | 1.2 |
| **Average** | 51.5 | 3.80 | 1.3 |

**Supplementary Figure 35. Mechanical properties of flexible free-standing MXene membranes. a,** Photo of the universal testing machine with a free-standing MXene membrane (size of 30 mm × 10 mm); **b,** Stress-strain curves of four MXene membranes with thickness around 2 μm. The table under the figure shows the testing data.

**Supplementary Note 8:** For the mechanical testing, the MXene membranes were cut into strips (30 mm×10 mm). The tensile tests were performed at a loading rate of 1 mm $min^{-1}$ at room temperature.

**Supplementary Table 1** EDX analysis of MXene membranes.

| **MXene membrane** | Elemental EDX analysis (atomic ratio) | | | | |
|---|---|---|---|---|---|
| | **Ti** | **C** | **O** | **F** | **Al** |
| M1 | 3.00 | 2.20 | 2.32 | 0.79 | 0.04 |
| M2 | 3.00 | 2.15 | 2.47 | 0.88 | 0.05 |
| M3 | 3.00 | 1.95 | 2.54 | 0.87 | 0.04 |
| Average | 3.00<br>(35.6 %) [#] | 2.10<br>(24.9 %) | 2.44<br>(29.0 %) | 0.85<br>(10.1 %) | 0.04<br>(0.47 %) |

[#] Percentage of atoms

**Supplementary Table 2** XPS analysis of MXene membranes.

| **MXene membrane** | **XPS-Elements (Atomic ratio)** | | | | |
|---|---|---|---|---|---|
| | Ti | $C^{\#}$ | O | F | Li |
| M1 | 3.00 | 4.10 | 2.02 | 0.92 | 0.06 |
| M2 | 3.00 | 5.40 | 2.52 | 0.99 | 0.04 |
| M3 | 3.00 | 3.80 | 1.93 | 0.94 | 0.07 |
| Average | **3.00 (28.3%)** | **4.43 (41.8%)** | **2.16 (20.4%)** | **0.95 (8.9%)** | **0.057 (0.54%)** |

$^{\#}$XPS results show a relatively high content of C, which may be due to the contamination caused by the ambient atmosphere and/or as a result of processing[10]. Because of the use of LiF/HCl as the etchants, a low concentration (0.54 at.%) of lithium remained in the MXene membrane, which can be ignored.

**Supplementary Table 3** Fits of the oxygen regions from XPS results of MXene membranes.

| MXene membrane | Terminations (at.%) | |
|---|---|---|
| | =O | -OH |
| M1 | 1 | 0.39 |
| M2 | 1 | 0.34 |
| M3 | 1 | 0.37 |
| Average | **1** | **0.37** |

**Supplementary Table 4** Separation performance of a 2-μm-thick MXene membrane in both single gas and equimolar mixed gas permeations.

| | | Performance of MXene membrane | | | | | |
|---|---|---|---|---|---|---|---|
| | | Single gas | | | Mixed gas | | |
| Gas i/j | Knudsen constant | Permeability (Barrer) | | Ideal selectivity | Permeability (Barrer) | | Separation factor |
| | | i | j | | i | j | |
| $H_2/CO_2$ | 4.7 | 2402.3 | 10.1 | 238 | 2226.6 | 14.2 | 167 |
| $H_2/N_2$ | 3.7 | 2402.3 | 18.7 | 129 | 1975.9 | 25.4 | 78 |
| $H_2/CH_4$ | 2.8 | 2402.3 | 3.1 | 780 | 1930.6 | 6.3 | 324 |
| $H_2/C_3H_6$ | 4.6 | 2402.3 | 1.9 | 1258 | 2311.9 | 3.1 | 782 |
| $H_2/C_3H_8$ | 4.7 | 2402.3 | 1.2 | 2024 | 2356.8 | 2.3 | 1102 |

**Supplementary Table 5** MD simulated data for single gas permeation through the MXene membrane. Corresponding snapshots are shown in Supplementary Fig. 26.

| **Gas** | **Single gas permeation MD Simulated Flux (Number of molecules ns$^{-1}$)** | | | | **Ideal Selectivity** | |
|---|---|---|---|---|---|---|
| | MD | Flux | Average | Standard deviation | $H_2/CO_2$ | $H_2/N_2$ |
| **$H_2$** | MD1# | 0.8 | 0.75 | 0.05 | 200 | 120 |
| | MD2 | 0.7 | | | | |
| | MD3 | 0.8 | | | | |
| | MD4 | 0.7 | | | | |
| **$CO_2$** | MD1 | 0 | 0.0038 | 0.0041 | | |
| | MD2 | 0 | | | | |
| | MD3 | 0.01 | | | | |
| | MD4 | 0.005 | | | | |
| **$O_2$** | MD1 | 0.0075 | 0.0071 | 0.0024 | | |
| | MD2 | 0.005 | | | | |
| | MD3 | 0.005 | | | | |
| | MD4 | 0.0109 | | | | |
| **$N_2$** | MD1 | 0.01 | 0.0063 | 0.0022 | | |
| | MD2 | 0.005 | | | | |
| | MD3 | 0.005 | | | | |
| | MD4 | 0.005 | | | | |

#The number k (k = 1 to 4) in MDk refers to the k$^{th}$ independent MD simulation. Same for Supplementary Table 6.

**Supplementary Table 6** MD simulated data for mixed gas permeation through the MXene membrane. Corresponding snapshots are shown in main text Fig. 2d.

| Gas i/j | Mixed gas permeation MD Simulated Flux (Number of molecules $ns^{-1}$) | | | | | | | Selectivity |
|---|---|---|---|---|---|---|---|---|
| | MD | i | Average | Standard deviation | j | Average | Standard deviation | |
| **$H_2/CO_2$** | MD1# | 0.5 | 0.68 | 0.16 | 0.0067 | 0.0042 | 0.0016 | 162 |
| | MD2 | 0.7 | | | 0.0033 | | | |
| | MD3 | 0.9 | | | 0.0033 | | | |
| | MD4 | 0.6 | | | 0.0033 | | | |
| **$H_2/N_2$** | MD1 | 0.7 | 0.75 | 0.087 | 0.01 | 0.0083 | 0.0017 | 90 |
| | MD2 | 0.9 | | | 0.007 | | | |
| | MD3 | 0.7 | | | 0.01 | | | |
| | MD4 | 0.7 | | | 0.007 | | | |

**Supplementary Table 7** MD simulated data for single gas permeation through the MXene membrane with the composition of $Ti_3C_2F_2$.

| **Gas** | **Single gas permeation MD Simulated Flux (Number of molecule $ns^{-1}$)** | | | | **Ideal Selectivity** |
|---|---|---|---|---|---|
| | MD | Flux | Average | Standard deviation | $H_2/CO_2$ |
| **$H_2$** | MD1 | 0.97 | 0.85 | 0.0086 | 171 |
| | MD2 | 0.60 | | | |
| | MD3 | 0.97 | | | |
| | MD4 | 0.87 | | | |
| **$CO_2$** | MD1 | 0.0059 | 0.005 | 0.00064 | |
| | MD2 | 0.0041 | | | |
| | MD3 | 0.0050 | | | |
| | MD4 | 0.0050 | | | |

**Supplementary Table 8** Gas separation performance of several MXene membranes produced from different batches under the same conditions, which exhibit high reproducibility.

| MXene membrane | Permeability (Barrer) | | | | Selectivity | Average selectivity | Standard deviation of selectivity |
|---|---|---|---|---|---|---|---|
| | $H_2$ | Average | $CO_2$ | Average | $H_2/CO_2$ | | |
| **M1** | 2226.6 | 2242.4 | 14.2 | 15.5 | 167.0 | 155.9 | 15.3 |
| **M2** | 2035.3 | | 16.4 | | 138.9 | | |
| **M3** | 2542.2 | | 17.8 | | 142.8 | | |
| **M4** | 2165.4 | | 13.4 | | 174.9 | | |

**Supplementary Table 9** Detailed test conditions of the data points shown in main text Fig. 3d.

| | Membrane Material | Thickness (μm) | Temp. (°C) | $H_2/CO_2$ Ratio | Permeability (Barrer) | Selectivity ($H_2/CO_2$) | Ref. |
|---|---|---|---|---|---|---|---|
| **1** | Si 400 | 0.03 | 200 | 50/50 | 179.1 | 7.5 | 14 |
| | Si 600 | 0.03 | 200 | 50/50 | 44.8 | 71 | 14 |
| **2** | Silicon Carbide | 2 | 200 | N.A. | 53.1 | 50 | 15 |
| **3** | ZSM-5/Silicalite | 9.5 | 450 | 50/50 | 3581.5 | 25.3 | 16 |
| **4** | Modified MFI | 2 | 500 | 50/50 | 59.0 | 45.6 | 17 |
| **5** | CMS | N.A. | 25 | N.A. | 300.0 | 0.92 | 18 |
| | CMS | N.A. | 25 | | 1.6 | 15 | 18 |
| **6** | PBI | N.A. | 35 | 50/50 | 2.9 | 7.1 | 19 |
| **7** | CAU-1 | 4 | 25 | N.A. | 1289.6 | 12.3 | 20 |
| **8** | ZIF-8 | 6 | 30 | 50/50 | 2829.9 | 4.6 | 21 |
| **9** | ZIF-7 | 2 | 220 | 50/50 | 271.6 | 13.6 | 22 |
| **10** | KUUST-1 | 60 | 25 | 50/50 | 179104.5 | 6.8 | 23 |
| **11** | SIM-1 | 25 | 30 | N.A. | 6119.4 | 2.3 | 24 |
| **12** | COF-MOF | 97.2 | 25 | N.A. | 110000.0 | 13.5 | 25 |
| **13** | GO | 0.0018 | 20 | 50/50 | 0.67 | 2100 | 26 |
| | GO | 0.009 | 20 | 50/50 | 3.1 | 3400 | 26 |
| | GO | 0.018 | 20 | 50/50 | 5.6 | 2300 | 26 |
| **14** | GO | 0.003-0.01 | 130 | N.A. | ~35 | ~30 | 27 |
| | GO | 0.003-0.01 | 140 | | ~44 | 40 | 27 |
| **15** | ZIF-8/GO | 20 | 250 | 50/50 | ~7761.2 | 14.9 | 28 |
| **16** | EFDA−GO | N.A. | 25 | 50/50 | 840.0 | 33 | 29 |
| | EFDA−GO | N.A. | 25 | | 1200.0 | 29 | 29 |
| **17** | 1T $MoS_2$ | 1 | 25 | 50/50 | 1329.0 | 7.6 | 30 |
| | 2H $MoS_2$ | 1 | 25 | 50/50 | 1740.0 | 6 | 30 |
| **18** | $MoS_2$ | 0.017 | 35 | N.A. | 466.5 | 3.4 | 31 |
| | $MoS_2$ | 0.035 | 35 | | 246.5 | 3.7 | 31 |
| | $MoS_2$ | 0.06 | 35 | | 146.8 | 4.4 | 31 |

| | | | | | | | |
|---|---|---|---|---|---|---|---|
| **19** | 2D ZIFs | N.A. | 25 | 50/50 | 302.0 | 172 | 32 |
| | 2D ZIFs | | 25 | 50/50 | 194.7 | 191 | 32 |
| | 2D ZIFs | | 25 | 50/50 | 254.6 | 261 | 32 |
| | 2D ZIFs | | 25 | 50/50 | 206.0 | 191 | 32 |
| | 2D ZIFs | | 25 | 50/50 | 119.6 | 98 | 32 |
| **20** | 2D MOFs | 0.04 | 20 | 50/50 | 28.6 | 245 | 33 |
| | 2D MOFs | 0.04 | 40 | 50/50 | 35.2 | 225 | 33 |
| | 2D MOFs | 0.04 | 20 | 20/80 | 31.6 | 167 | 33 |
| | 2D MOFs | 0.04 | 20 | 50/50 | 15.7 | 215 | 33 |
| | 2D MOFs | 0.04 | 40 | 50/50 | 17.2 | 194 | 33 |
| **21** | MXene | 1.1 | 25 | 50/50 | 3202.3 | 62 | This work |
| | MXene | 2 | 25 | 50/50 | 2226.6 | 167 | This work |
| | MXene | 3.2 | 25 | 50/50 | 1473.3 | 174 | This work |
| | MXene | 5.1 | 25 | 50/50 | 1302.7 | 200 | This work |

For the unknown membrane thickness, permeance is converted to permeability assuming the thickness of 0.1 μm[25,32].

**Supplementary Note 9:** Supplementary Table 9 and main text Fig. 3d summarized the latest various membranes in separation of $H_2/CO_2$, including zeolites, MOFs, ZIFs, GO and $MoS_2$. In similar conditions, MXene membranes display outstanding separation performance in terms of the permeability and selectivity.

**Supplementary References**


1. Naguib M., Mochalin V. N., Barsoum M. W., Gogotsi Y. 25th anniversary article: MXenes: a new family of two-dimensional materials. *Adv. Mater.*, **26,** 992-1005 (2014).
2. Naguib M. *et al.* Two-dimensional nanocrystals produced by exfoliation of $Ti_3AlC_2$. *Adv. Mater.*, **23,** 4248-4253 (2011).
3. Barsoum M. W. *MAX phases: properties of machinable ternary carbides and nitrides*. John Wiley & Sons, (2013).
4. Lukatskaya M. R. *et al.* Cation intercalation and high volumetric capacitance of two-dimensional titanium carbide. *Science,* **341,** 1502-1505 (2013).
5. Barsoum M. W. The $M_{N+1}AX_N$ phases: A new class of solids: Thermodynamically stable nanolaminates. *Prog. Solid State Chem.*, **28,** 201-281 (2000).
6. Sun Z., Music D., Ahuja R., Li S., Schneider J. M. Bonding and classification of nanolayered ternary carbides. *Phys. Rev. B,* **70,** 092102 (2004).
7. Hope M. A. *et al.* NMR reveals the surface functionalisation of $Ti_3C_2$ MXene. *Phys. Chem. Chem. Phys.*, **18,** 5099-5102 (2016).
8. Lipatov A. *et al.* Effect of Synthesis on Quality, Electronic Properties and Environmental Stability of Individual Monolayer $Ti_3C_2$ MXene Flakes. *Adv. Electron. Mater.*, **2,** 1600255 (2016).
9. Cao Y. *et al.* Enhanced thermal properties of poly (vinylidene fluoride) composites with ultrathin nanosheets of MXene. *RSC Adv.*, **7,** 20494-20501 (2017).
10. Halim J. *et al.* X-ray photoelectron spectroscopy of select multi-layered transition metal carbides (MXenes). *Appl. Surf. Sci.*, **362,** 406-417 (2016).
11. Wang L. *et al.* Synthesis and electrochemical performance of $Ti_3C_2Tx$ with hydrothermal process. *Electron. Mater. Lett.*, **12,** 702-710 (2016).
12. Han M. *et al.* $Ti_3C_2$ MXenes with Modified Surface for High-Performance Electromagnetic Absorption and Shielding in the X-Band. *ACS Appl. Mater. Interfaces,* **8,** 21011-21019 (2016).
13. Nair R. R., Wu H. A., Jayaram P. N., Grigorieva I. V., Geim A. K. Unimpeded permeation of water through helium-leak-tight graphene-based membranes. *Science,* **335,** 442-444 (2012).
14. De Vos R. M., Verweij H. High-selectivity, high-flux silica membranes for gas separation. *Science*, **279,** 1710-1711 (1998).
15. Elyassi B., Sahimi M., Tsotsis T. T. Silicon carbide membranes for gas separation applications. *J. Membr. Sci.*, **288,** 290-297 (2007).
16. Wang H., Lin Y. Synthesis and modification of ZSM-5/silicalite bilayer membrane with improved hydrogen separation performance. *J. Membr. Sci.*, **396,** 128-137 (2012).

17. Tang Z., Dong J., Nenoff T. M. Internal surface modification of MFI-type zeolite membranes for high selectivity and high flux for hydrogen. *Langmuir*, **25,** 4848-4852 (2009).

18. Shekhawat D., Luebke D. R., Pennline H. W. A review of carbon dioxide selective membranes. *US DOE***,** 9-11 (2003).

19. Yang T., Xiao Y., Chung T.-S. Poly-/metal-benzimidazole nano-composite membranes for hydrogen purification. *Energy Environ. Sci.*, **4,** 4171-4180 (2011).

20. Zhou S. *et al.* Development of hydrogen-selective CAU-1 MOF membranes for hydrogen purification by 'dual-metal-source'approach. *Int. J. Hydrogen Energy*, **38,** 5338-5347 (2013).

21. Zhang X. *et al.* New membrane architecture with high performance: ZIF-8 membrane supported on vertically aligned ZnO nanorods for gas permeation and separation. *Chem. Mater.*, **26,** 1975-1981 (2014).

22. Li Y., Liang F., Bux H., Yang W., Caro J. Zeolitic imidazolate framework ZIF-7 based molecular sieve membrane for hydrogen separation. *J. Membr. Sci.*, **354,** 48-54 (2010).

23. Guo H., Zhu G., Hewitt I. J., Qiu S. "Twin Copper Source" Growth of Metal-Organic Framework Membrane: $Cu_3(BTC)_2$ with High Permeability and Selectivity for Recycling $H_2$. *J. Am. Chem. Soc.*, **131,** 1646-1647 (2009).

24. Aguado S. *et al.* Facile synthesis of an ultramicroporous MOF tubular membrane with selectivity towards $CO_2$. *New J. Chem.*, **35,** 41-44 (2011).

25. Fu J. *et al.* Fabrication of COF-MOF Composite Membranes and Their Highly Selective Separation of $H_2/CO_2$. *J. Am. Chem. Soc.*, **138,** 7673-7680 (2016).

26. Li H. *et al.* Ultrathin, molecular-sieving graphene oxide membranes for selective hydrogen separation. *Science*, **342,** 95-98 (2013).

27. Kim H. W. *et al.* Selective gas transport through few-layered graphene and graphene oxide membranes. *Science*, **342,** 91-95 (2013).

28. Huang A., Liu Q., Wang N., Zhu Y., Caro J. Bicontinuous zeolitic imidazolate framework ZIF-8@GO membrane with enhanced hydrogen selectivity. *J. Am. Chem. Soc.*, **136,** 14686-14689 (2014).

29. Shen J. *et al.* Subnanometer two-dimensional graphene oxide channels for ultrafast gas sieving. *ACS nano*, **10,** 3398-3409 (2016).

30. Achari A., Sahana S., Eswaramoorthy M. High performance $MoS_2$ membranes: effects of thermally driven phase transition on $CO_2$ separation efficiency. *Energy Environ. Sci.*, **9,** 1224-1228 (2016).

31. Wang D., Wang Z., Wang L., Hu L., Jin J. Ultrathin membranes of single-layered $MoS_2$ nanosheets for high-permeance hydrogen separation. *Nanoscale*, **7,** 17649-17652 (2015).

32. Peng Y. *et al.* Metal-organic framework nanosheets as building blocks for molecular sieving membranes. *Science*, **346,** 1356-1359 (2014).

33. Wang X. *et al.* Reversed thermo-switchable molecular sieving membranes composed of two-dimensional metal-organic nanosheets for gas separation. *Nat. Commun.*, **8,** 14460 (2017).
